# Whole genome sequence association analysis of fasting glucose and fasting insulin levels in diverse cohorts from the NHLBI TOPMed program

**DOI:** 10.1038/s42003-022-03702-4

**Published:** 2022-07-28

**Authors:** Daniel DiCorpo, Sheila M. Gaynor, Emily M. Russell, Kenneth E. Westerman, Laura M. Raffield, Timothy D. Majarian, Peitao Wu, Chloé Sarnowski, Heather M. Highland, Anne Jackson, Natalie R. Hasbani, Paul S. de Vries, Jennifer A. Brody, Bertha Hidalgo, Xiuqing Guo, James A. Perry, Jeffrey R. O’Connell, Samantha Lent, May E. Montasser, Brian E. Cade, Deepti Jain, Heming Wang, Ricardo D’Oliveira Albanus, Arushi Varshney, Lisa R. Yanek, Leslie Lange, Nicholette D. Palmer, Marcio Almeida, Juan M. Peralta, Stella Aslibekyan, Abigail S. Baldridge, Alain G. Bertoni, Lawrence F. Bielak, Chung-Shiuan Chen, Yii-Der Ida Chen, Won Jung Choi, Mark O. Goodarzi, James S. Floyd, Marguerite R. Irvin, Rita R. Kalyani, Tanika N. Kelly, Seonwook Lee, Ching-Ti Liu, Douglas Loesch, JoAnn E. Manson, Ryan L. Minster, Take Naseri, James S. Pankow, Laura J. Rasmussen-Torvik, Alexander P. Reiner, Muagututi’a Sefuiva Reupena, Elizabeth Selvin, Jennifer A. Smith, Daniel E. Weeks, Huichun Xu, Jie Yao, Wei Zhao, Stephen Parker, Alvaro Alonso, Donna K. Arnett, John Blangero, Eric Boerwinkle, Adolfo Correa, L. Adrienne Cupples, Joanne E. Curran, Ravindranath Duggirala, Jiang He, Susan R. Heckbert, Sharon L. R. Kardia, Ryan W. Kim, Charles Kooperberg, Simin Liu, Rasika A. Mathias, Stephen T. McGarvey, Braxton D. Mitchell, Alanna C. Morrison, Patricia A. Peyser, Bruce M. Psaty, Susan Redline, Alan R. Shuldiner, Kent D. Taylor, Ramachandran S. Vasan, Karine A. Viaud-Martinez, Jose C. Florez, James G. Wilson, Robert Sladek, Stephen S. Rich, Jerome I. Rotter, Xihong Lin, Josée Dupuis, James B. Meigs, Jennifer Wessel, Alisa K. Manning

**Affiliations:** 1 Department of Biostatistics, Boston University School of Public Health, Boston, MA 02118 USA; 2 Department of Biostatistics, Harvard T.H. Chan School of Public Health, Boston, MA 02115 USA; 3 Department of Human Genetics, Graduate School of Public Health, University of Pittsburgh, Pittsburgh, PA 15261 USA; 4 Clinical and Translational Epidemiology Unit, Mongan Institute, Massachusetts General Hospital, Boston, MA 02114 USA; 5 Metabolism Program, The Broad Institute of MIT and Harvard, Cambridge, MA 02124 USA; 6 Department of Medicine, Harvard Medical School, Boston, MA 02115 USA; 7 Department of Genetics, University of North Carolina, Chapel Hill, NC 27599 USA; 8 Department of Epidemiology, University of North Carolina at Chapel Hill, Chapel Hill, NC 27514 USA; 9 Department of Biostatistics, University of Michigan, Ann Arbor, MI 48109 USA; 10 Department of Epidemiology, Human Genetics, and Environmental Sciences, School of Public Health, The University of Texas Health Science Center at Houston, Houston, TX 77030 USA; 11 Human Genetics Center, Department of Epidemiology, Human Genetics, and Environmental Sciences, School of Public Health, The University of Texas Health Science Center at Houston, Houston, TX 77030 USA; 12 Cardiovascular Health Research Unit, University of Washington, Seattle, WA 98101 USA; 13 Department of Medicine, University of Washington, Seattle, WA 98101 USA; 14 Ryals School of Public Health, University of Alabama at Birmingham, Birmingham, AL 35294 USA; 15 The Institute for Translational Genomics and Population Sciences, Department of Pediatrics, The Lundquist Institute for Biomedical Innovation at Harbor-UCLA Medical Center, Torrance, CA 90502 USA; 16 Division of Endocrinology, Diabetes, and Nutrition, University of Maryland School of Medicine, Baltimore, MD 21201 USA; 17 Program for Personalized and Genomic Medicine, University of Maryland School of Medicine, Baltimore, MD 21201 USA; 18 Division of Sleep and Circadian Disorders, Brigham and Women’s Hospital, Boston, MA 02115 USA; 19 Division of Sleep Medicine, Harvard Medical School, Boston, MA 02115 USA; 20 Program in Medical and Population Genetics, The Broad Institute of MIT and Harvard, Cambridge, MA 02124 USA; 21 Department of Biostatistics, University of Washington, Seattle, WA 98195 USA; 22 Department of Computational Medicine & Bioinformatics, University of Michigan, Ann Arbor, MI 48109 USA; 23 GeneSTAR Research Program, Johns Hopkins University School of Medicine, Baltimore, MD 21287 USA; 24 Department of Medicine, Anschutz Medical Campus, University of Colorado Denver, Aurora, CO 80045 USA; 25 Department of Biochemistry, Wake Forest School of Medicine, Winston-Salem, NC 27157 USA; 26 Department of Human Genetics and South Texas Diabetes and Obesity Institute, University of Texas Rio Grande Valley School of Medicine, Brownsville and Edinburg, TX 78539 USA; 27 23andMe, Sunnyvale, CA 94086 USA; 28 Department of Preventive Medicine, Northwestern University Feinberg School of Medicine, Chicago, IL 60611 USA; 29 Department of Epidemiology & Prevention, Wake Forest School of Medicine, Winston-, Salem, NC 27157 USA; 30 Department of Epidemiology, School of Public Health, University of Michigan, Ann Arbor, MI 48109 USA; 31 Department of Epidemiology, Tulane University School of Public Health and Tropical Medicine, New Orleans, LA 70112 USA; 32 Psomagen, Inc, Rockville, MD 20850 USA; 33 Department of Medicine, Division of Endocrinology, Diabetes and Metabolism, Cedars-Sinai Medical Center, Los Angeles, CA 90048 USA; 34 Cardiovascular Health Research Unit, University of Washington, Seattle, WA 98195 USA; 35 Department of Medicine, University of Washington, Seattle, WA 98195 USA; 36 Institute for Genome Sciences, University of Maryland School of Medicine, Baltimore, MD 21201 USA; 37 Department of Medicine, University of Maryland School of Medicine, Baltimore, MD 21201 USA; 38 Brigham and Women’s Hospital, Boston, MA 02115 USA; 39 Ministry of Health, Government of Samoa, Apia, Samoa; 40 Division of Epidemiology and Community Health, School of Public Health, University of Minnesota, Minneapolis, MN 55454 USA; 41 Department of Epidemiology, University of Washington, Seattle, WA 98195 USA; 42 Lutia i Puava ‘ae Mapu i Fagalele, Apia, Samoa; 43 Department of Epidemiology, Johns Hopkins Bloomberg School of Public Health, Baltimore, MD 21287 USA; 44 Survey Research Center, Institute for Social Research, University of Michigan, Ann Arbor, MI USA; 45 Department of Biostatistics, Graduate School of Public Health, University of Pittsburgh, Pittsburgh, PA 15261 USA; 46 Department of Human Genetics, University of Michigan, Ann Arbor, MI 48109 USA; 47 Department of Epidemiology, Rollins School of Public Health, Emory University, Atlanta, GA 30322 USA; 48 College of Public Health, University of Kentucky, Lexington, KY 40506 USA; 49 Department of Medicine, University of Mississippi Medical Center, Jackson, MS 39211 USA; 50 National Heart Lung and Blood Institute and Boston University’s Framingham Heart Study, Framingham, MA 01702 USA; 51 Division of Public Health Sciences, Fred Hutchinson Cancer Research Center, Seattle, WA 98109 USA; 52 Center for Global Cardiometabolic Health (CGCH), Boston, MA 02215 USA; 53 International Health Institute and Department of Epidemiology, Brown University School of Public Health, Providence, RI 02912 USA; 54 Geriatrics Research and Education Clinical Center, Baltimore VA Medical Center, Baltimore, MD 21201 USA; 55 Department of Health Services, University of Washington, Seattle, WA 98101 USA; 56 Division of Pulmonary, Critical Care, and Sleep Medicine, Beth Israel Deaconess Medical Center, Boston, MA 02115 USA; 57 Program for Personalized and Genomic Medicine, University of Maryland School of Medicine, Baltimore, MD 21231 USA; 58 Evans Department of Medicine, Section of Preventive Medicine and Epidemiology, Boston University School of Medicine, Boston, MA 02118 USA; 59 Evans Department of Medicine, Whitaker Cardiovascular Institute and Cardiology Section, Boston University School of Medicine, Boston, MA 02118 USA; 60 Illumina Laboratory Services, Illumina, Inc, San Diego, CA 92122 USA; 61 Center for Genomic Medicine and Diabetes Unit, Massachusetts General Hospital, Boston, MA 02114 USA; 62 Division of Cardiovascular Medicine, Beth Israel Deaconess Medical Center, Boston, MA 02115 USA; 63 Department of Human Genetics, McGill University, Montreal, Montreal, Quebec H3A 0G1 Canada; 64 Department of Medicine, McGill University, Montreal, Montreal, Quebec H3A 0G1 Canada; 65 Center for Public Health Genomics, University of Virginia, Charlottesville, VA 22908 USA; 66 Division of General Internal Medicine, Massachusetts General Hospital, Boston, MA 02114 USA; 67 Department of Epidemiology, Fairbanks School of Public Health, Indiana University, IN 46202 USA; 68 Department of Medicine, School of Medicine, Indiana University, IN 46202 USA; 69 Diabetes Translational Research Center, Indiana University, IN 46202 USA

**Keywords:** Sequencing, Genetics research, Diabetes, Quantitative trait

## Abstract

The genetic determinants of fasting glucose (FG) and fasting insulin (FI) have been studied mostly through genome arrays, resulting in over 100 associated variants. We extended this work with high-coverage whole genome sequencing analyses from fifteen cohorts in NHLBI’s Trans-Omics for Precision Medicine (TOPMed) program. Over 23,000 non-diabetic individuals from five race-ethnicities/populations (African, Asian, European, Hispanic and Samoan) were included. Eight variants were significantly associated with FG or FI across previously identified regions *MTNR1B, G6PC2, GCK, GCKR* and *FOXA2*. We additionally characterize suggestive associations with FG or FI near previously identified *SLC30A8, TCF7L2*, and *ADCY5* regions as well as *APOB, PTPRT*, and *ROBO1*. Functional annotation resources including the Diabetes Epigenome Atlas were compiled for each signal (chromatin states, annotation principal components, and others) to elucidate variant-to-function hypotheses. We provide a catalog of nucleotide-resolution genomic variation spanning intergenic and intronic regions creating a foundation for future sequencing-based investigations of glycemic traits.

## Abstract (web summary)

This study of 23,000 non-diabetic individuals highlights loci associated with fasting glucose and fasting insulin in diverse cohorts with whole genome sequence data.

## Introduction

Type 2 diabetes (T2D) and insulin resistance are complex genetic conditions often characterized by disruptions of normal levels of fasting glucose (FG) and fasting insulin (FI)^1^. These traits are influenced by a spectrum of common to rare genetic variation^2–7^ with most evidence coming from genome-wide association studies (GWAS)^8,9^, exome arrays^2,3,6^, whole-exome sequencing^2^, and small samples of low-pass whole-genome sequencing (WGS)^4,10^. These efforts have found over 100, mostly common (minor allele frequency (MAF) > 0.05), variants associated with FG or FI, including those in non-coding and intergenic spaces^2–4,6,8,9^. We expand on these previous studies by assessing common, low frequency (MAF < 0.05), and rare (MAF < 0.01) variants through comprehensive WGS association analysis in diverse population cohorts in NHLBI’s Trans-Omics for Precision Medicine (TOPMed) program. The current study aims to better understand the variants at GWAS loci through multiple approaches including discovery and fine-mapping in both coding and non-coding regions as well as aggregate rare variant testing using both protein-coding variants and intergenic variants. In addition, we explore ancestry-specific results through our four included race/ethnicities and one population group: African, Asian, European, Hispanic, and Samoan, respectively. Finally, we characterize all reported regions with annotations including chromatin states, annotation principal components (PCs), expression quantitative trait loci (eQTL), and others from recent annotation accumulation efforts including the Diabetes Genome Atlas (DGA).

## Results

### Phenotypes and genotypes in the NHLBI TOPMed program

We identified and characterized common and rare variants associated with FG and (natural log-transformed) FI through association tests using WGS data from fifteen cohorts in TOPMed (Supplementary Table 1). As in prior quantitative trait analyses, we excluded individuals with diabetes (by glycemia or medication), resulting in a total sample size of 26,807 individuals with FG and 23,211 individuals with FI. This represents a diverse sample of four self-reported race/ethnicities and one population group including African, Asian, European, Hispanic, and Samoan, respectively, and our total sample was >40% non-European (Supplementary Tables 2–3). In addition to use of genetic ancestry adjustments in our models (see the “Methods” section), we used participant’s self-reported race/ethnicity to assign individuals to one of five groups for stratified analyses or inclusion as a covariate. Individuals were given a single label to infer their ancestry, but each group represents a diverse cross-section of race, culture, or admixture. Trait measures were harmonized across cohorts and assays and adjusted for self-reported race/ethnicity, study age, sex, and body mass index (BMI; Supplementary Tables 2–3, “Methods”). We assessed 60 M variants from the TOPMed Freeze 5b WGS data freeze for each trait using single-variant testing (minor allele count, MAC > 20) in pooled and race/ethnicity-specific approaches. We used a significance threshold of *P* < 1.0 × 10^−9^ as has been established for WGS studies including African ancestry^11^. We also separately report signals identified with *P* < 5.0 × 10^−8^ as suggestively associated with either trait. These suggestively associated signals are reported to characterize potential regions of interest with our available annotations for use in future higher-powered studies. We further performed rare variant testing (MAF < 0.01) using aggregate burden and SKAT tests in both gene centric and genetic region approaches. We computed 95% credible sets for each distinct common variant signal conditioned on any other identified signal at the locus (“Methods”, Supplementary Data 1). 99% credible sets are also reported for signals identified through the pooled analysis (Supplementary Data 2), with a median size of 12 variants. This is 20% smaller than a recent multi-ethnic GWAS of glycemic traits^12^ which reported a median 99% credible set size of 15.

### Whole-genome sequence significant associations with fasting glucose and insulin

We identified eight distinct variants significantly associated with FG or FI across five gene regions in the pooled race/ethnicity analysis (*P* < 1.0 × 10^−9^, Table 1). These include previously identified *MTNR1B, G6PC2, GCK, GCKR,* and *FOXA2* gene regions (Supplementary Data 3–4)*. MTNR1B* had a distinct secondary signal after conditioning on the lead variant. *G6PC2* had three distinct association signals, one of which was rare (MAF < 0.01), as determined by sequential conditional analysis. These distinct secondary and tertiary signals are also reported in Table 1 (locus-wide significance threshold of 1.0 × 10^−5^, “Methods”) and further described in the following sections. Manhattan and QQ plots for single variant analyses of FG and FI are shown in Supplementary Fig. 1.

**Table 1:** Distinct signals at loci significantly associated with glycemic traits FG and FI in TOPMed, *P* < 1 × 10^−9^.

Trait	Population	Nearest gene	MarkerID^a^	EA	rsID	Annotation	EAF	*P*-value	Beta	SE	Conditioned on
Fasting glucose	Pooled	*MTNR1B*	11:92975544:C:G	G	rs10830963	Intronic	0.24	9.1 × 10^−46^	0.07	0.01	
			11:92884161:G:A^b^	G	rs73560545	intronic	0.83	5.7 × 10^−6^	0.03	0.01	rs10830963
		*G6PC2*	2:168906638:T:C	C	rs560887	Intronic	0.82	6.8 × 10^−37^	0.07	0.01	
			2:168900420:A:G^b^	A	rs540524	2KB upstream	0.66	9.9 × 10^−14^	0.04	0.01	rs560887
			2:168907981:T:C ^c^	T	rs2232326	Missense	0.99	5.0 × 10^−6^	0.15	0.03	rs560887, rs540524
		*GCK*	7:44189469:C:T	T	rs1799884	2KB upstream	0.18	3.9 × 10^−28^	0.06	0.01	
		*GCKR*	2:27508073:T:C	C	rs1260326	Missense	0.65	6.1 × 10^−21^	0.04	0.01	
		*FOXA2*	20:22581688:A:AG	A	rs3833331	3′ UTR	0.85	5.4 × 10^−10^	0.04	0.01	
Fasting insulin	Pooled	*GCKR*	2:27508073:T:C	C	rs1260326	Missense	0.67	7.2 × 10^−13^	0.03	0.01	

*EA* effect allele, *EAF* effect allele frequency, *EU* European, *HS/L* Hispanic/Latinx.

^a^Chromosome, position(Hg38), reference allele, alternative allele of the genetic variant with the lowest *P*-value and highest posterior probability representing a distinct association at a locus.

^b^Indicates secondary signal.

^c^Indicates tertiary signal for association at significance level *P* < 1 × 10^−5^ and MAC > 20 after conditional analysis.

**Fig. 1:**
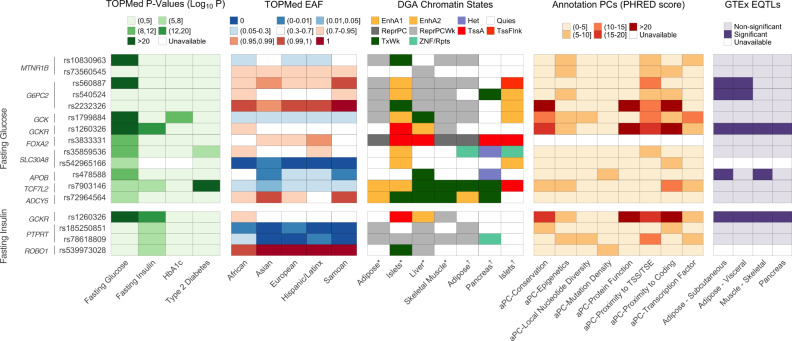
Characterization of significant and suggestive single-variant signals associated with fasting glucose and fasting insulin in TOPMed. TOPMed features of distinct, significant and suggestive signals associated with fasting glucose and fasting insulin (log-transformed) in pooled analysis. *P*-values (unconditional −log10-transformed) for glycemic and related traits (HbAa1c and type 2 diabetes) and effect allele frequency (with respect to the pooled analysis effect allele) across race/ethnicities in TOPMed are reported. Chromatin states at relevant tissues were drawn from two sets of experiments from DGA^46,47^; annotation PCs provide summaries of multi-faceted variant function; variants that are significant eQTLs in relevant tissues are denoted. EAF, effect allele frequency for TOPMed sample; EnhA1, Active Enhancer 1; EnhA2, Active Enhancer 2; Het, Heterochromatin; Quies, Quiescent/Low; ReprPC, Repressed PolyComb; ReprPCWk, Weak Repressed PolyComb; TssA, Active TSS; TssFlnk, Flanking TSS; TxWk, Weak Transcription; ZNF/Rpts, ZNF genes & repeats.

Our significantly associated variants replicate previous GWAS findings, which are summarized in Supplementary Data 3–4. We further characterize these variants in the context of sequencing and related available resources as summarized in Fig. 1. We used the Diabetes Epigenome Atlas (DGA) and TOPMed resources to provide functional annotations including chromatin states, annotation principal components (aPCs)^11^ that each provide a summary of related functional annotations via PCA (“Methods”), and expression quantitative trait loci (eQTL) from adipose, pancreas, liver and skeletal muscle. In addition to variant descriptions in Fig. 1, regional locus plots with tissue-specific annotations for reported loci in Supplementary Fig. 2, and associations of reported loci with related traits in Supplementary Fig. 3 and Supplementary Data 5. Selected regions are further described based on the data below.

*MTNR1B* intronic variant rs10830963 (*P* = 9.1 × 10^−46^) has been characterized as a strong signal for insulin secretion^13^; this variant is in a weak transcription chromatin state in islets, is a metabolite QTL for glucose^14,15^, and is a pancreatic-islet-specific eQTL associated with the expression of *MTNR1B*^16^. Identified after conditioning on the primary variant rs10830963 in the *MTNR1B* region, intronic variant rs73560545 occurs upstream of the primary signal and had a lowering effect on FG, in contrast to the primary signal which had a glucose-raising effect. While this is a well-known region in the context of these traits, this secondary variant at rs73560545 has not been previously identified in the reviewed literature (Supplementary Data 3–4).

The *GCKR* locus had a significant association with both FG and FI at rs1260326 (*P* = 6.1 × 10^−21^, 7.2 × 10^−13^, respectively) with functional activity suggested by its relatively high aPC-Epigenetics and aPC-Transcription-Factor values. This variant is also an eQTL and pQTL associated with many genes and proteins, most relevantly with insulin^17^. *GCKR* and rs1260326 have been previously described in previous studies for both traits (Supplementary Data 3–4).

The *FOXA2* locus has also been previously found to be associated with glycemic traits, regulating gene expression for glucose sensing in pancreatic beta cells^18^. We observe one FG-associated signal at rs3833331 (*P* = 5.4 × 10–10), a variant moderately linked to previously identified *FOXA2* lead variants rs6048205 and rs6048216, and based on conditional analysis is likely the same signal. The variant rs3833331 is in the 3′ UTR of the gene and classified as a CAGE promoter, GeneHancer, and SuperEnhancer. It is in an active TSS chromatin state for both pancreas and islets. Our identified variant rs3833331 is frequent in African individuals, while it has relatively low frequency in both European and Hispanic race/ethnicities.

Rare variant aggregate testing performed using both gene-centric and genetic region approaches identified one significantly associated region with FG at the known *G6PC2* locus, described in the next section (Supplementary Data 6–7). No rare variant aggregate signals were found to be associated with FI that were not composed mostly of singletons (Supplementary Data 8–9). Manhattan plots for region-based rare variant aggregate analysis in Supplementary Fig. 4.

### Refinement of the multi-allelic associations at the known G6PC2 locus

**Fig. 2:**
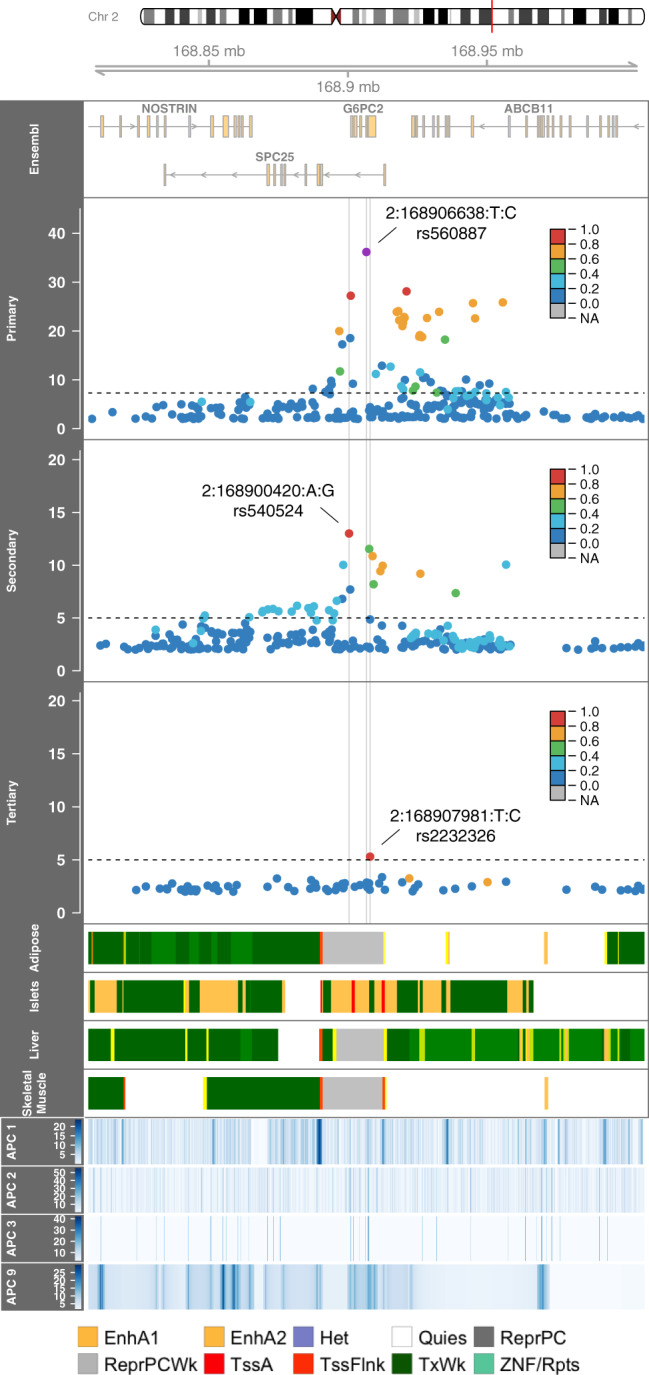
Regional investigation of three conditionally significant signals associated with fasting glucose in the *G6PC2* locus in TOPMed. Regional association plot of −log10 *P* values by genomic position for sequential conditional single-variant analyses. The linkage disequilibrium (*r*^2^) between the primary signal (rs560887, 2:168906638:T:C), as defined by the highest posterior probability, and variants in the region for each panel as calculated in TOPMed is indicated in the colors of the points. The chromatin states at four relevant tissues^47^ and annotation PCs are provided across the region. APC1, APC Epigenetics, APC2, APC Conservation, APC3, APC Protein, APC9, APC Distance to TSS/TSE; EnhA1, Active Enhancer 1; EnhA2, Active Enhancer 2; Het, Heterochromatin; Quies, Quiescent/Low; ReprPC, Repressed PolyComb; ReprPCWk, Weak Repressed PolyComb; TssA, Active TSS; TssFlnk, Flanking TSS; TxWk, Weak Transcription; ZNF/Rpts, ZNF genes & repeat.

At the known FG and T2D-associated *G6PC2* locus^2,3^, we observed several previously identified variant associations with FG (Fig. 2). In single variant analyses, we identified three distinct association signals, the third of which was a previously identified association after conditioning on two previously reported common GWAS variants, rs560887 (primary signal, *P* = 6.8 × 10^−37^) and rs540524 (secondary signal, *P* = 9.9 × 10^−14^). The rare missense variant rs2232326 (tertiary signal, *P* = 5.0 × 10^−6^) is annotated^19^ by the aPCs as disruptive and likely damaging, with a score falling in the top 7% distribution of the aPC representing “protein function” (aPC-Protein Function = 31.5, top 7% genome-wide). In addition, rs2232326 appears to be highly conserved, with a score falling in the top 0.13% of the distribution of an aPC representing “conservation” (aPC-Conservation = 28.8, top 0.13% genome-wide). The genomic region surrounding rs2232326 is annotated to be in a weakly transcribed chromatin state, relative to the genome, in islets and this variant is near the transcription end site (Fig. 2). The frequency of the C allele at rs2232326 was <0.01 in all race/ethnicity groups except for the Asian group where the frequency was 0.03 (gnomAD: East Asian AF = 0.05, Overall AF = 0.01). In aggregate gene-centric testing of all 75 rare missense variants in *G6PC2*, this previously identified rare (MAF = 0.01*)* variant rs2232326, along with variant rs2232323 (MAF = 0.01), contributed the most to the significant association test statistic (*P*_Burden,1,1_ = 1.4 × 10^−10^, Supplementary Data 6).

Given the multiple distinct signals at *G6PC2*, we performed a haplotype analysis to evaluate the contribution of rare variants and identify allele-specific effects. We extended the haplotype analysis of Mahajan et al.^3^ (rs560887, rs138726309, rs2232323, rs492594) to include our secondary (rs540524) and tertiary (rs2232326) signals. Our secondary signal is in moderate linkage (*r*^2^ = 0.58) to the previously haplotyped rs492594 and the effect allele A has a glucose-raising effect in both marginal and conditional analyses (Supplementary Table 4 and Table 1). We observed consistent direction of effects as the previous haplotype analysis, demonstrating the reliability of associations identified in the present TOPMed sample. Both haplotypes containing the C allele of the tertiary signal at rs2232326, the variant with the largest effect size included in the analysis, had glucose-lowering effects. The largest glucose-lowering effects at *G6PC2* were observed at the two haplotypes each carrying a rare allele: rs2232326 (rs560887-C, rs138726309-C, rs2232323-A, rs492594-C, rs540524-G, rs2232326-C, Beta = −0.15 +/− 0.00002) and rs2232323 (rs560887-T, rs138726309-C, rs2232323-C, rs492594-G, rs540524-A, rs2232326-T, Beta = −0.11+/−0.00008, Supplementary Table 4).

### Additional suggestive associations with fasting glucose and insulin

We further report twelve distinct variants suggestively associated with FG or FI across ten gene regions in the pooled race/ethnicity analysis and ancestry-specific analyses (*P* < 5.0 × 10^−8^; Table 2). These include previously identified *SLC30A8, TCF7L2*, and *ADCY5* gene regions (Supplementary Data 3–4). Other regions not previously identified include *APOB, PTPRT, ROBO1* and those described in the ancestry-specific section below. *SLC30A8* and *PTPRT* have distinct secondary signals identified through conditional analysis, which are also reported in Table 2 (locus-wide significance threshold *P* < 1.0 × 10^−5^). We outline these suggestive signals and the corresponding gene regions below to provide annotation and description and to provide context for investigation of these signals in future, larger studies.

**Table 2:** Distinct signals at loci suggestively associated with glycemic traits FG and FI in TOPMed, *P* < 5 × 10^−8^.

Trait	Population	Nearest gene	MarkerID^a^	EA	rsID	Annotation	EAF	*P*-value	Beta	SE	Conditioned on
Fasting glucose	Pooled	*SLC30A8*	8:117179236:C:T	C	rs35859536	2.5KB downstream	0.74	1.0 × 10^−9^	0.03	0.01	
			8:117258547:C:T^b^	T	rs542965166	Intronic	0.001	1.9 × 10^−6^	0.50	0.11	rs35859536
		*APOB*	2:21074277:A:G	A	rs478588	30KB upstream	0.29	2.9 × 10^−9^	0.03	0.01	
		*TCF7L2*	10:112998590:C:T	T	rs7903146	Intronic	0.25	2.0 × 10^−8^	0.03	0.01	
		*ADCY5*	3:123335923:A:C	A	rs72964564	Intronic	0.80	2.8 × 10^−8^	0.03	0.01	
	HS/L	*HS6ST3*	13:96407609:A:G	G	rs1328056	Intronic	0.02	3.6 × 10^−8^	0.33	0.06	
		*CTD-2199C04.4*	5:10169711:T:C	C	rs13361160	Intergenic	0.45	3.1 × 10^−8^	0.10	0.02	
Fasting insulin	Pooled	*PTPRT*	20:42752773:G:A	A	rs185250851	Intronic	0.002	2.1 × 10^−8^	0.30	0.05	
			20:43230137:C:T^b^	T	rs78618809	Intergenic	0.02	5.9 × 10^−6^	0.08	0.02	rs185250851
		*ROBO1*	3:79812347:C:A	C	rs539973028	44KB upstream	0.99	4.7 × 10^−8^	0.51	0.09	
	EU	*LINC00704, LINC00705*	10:4656482:GAAAAT:G	G	rs775018107	ncRNA intronic	0.002	4.5 × 10^−8^	0.33	0.06	
	Samoan	*RP11/IGSF11*	3:118656074:T:G^c^	G	rs117592405	ncRNA intronic	0.01	3.3 × 10^−8^	0.80	0.14	

*EA* effect allele, *EAF* effect allele frequency, *EU* European, *HS/L* Hispanic/Latinx.

^a^Chromosome, position(Hg38), reference allele, alternative allele of the genetic variant with the lowest *P*-value and highest posterior probability representing a distinct association at a locus.

^b^Indicates secondary signal for association at significance level *P* < 1 × 10^−5^ and MAC > 20 after conditional analysis.

^c^This signal was not replicated (Supplementary Table 7).

In the *ADCY5* region, variant rs72964564 (*P* = 2.8 × 10^−8^*)* showed suggestive association with FG and is highly linked (*r*^2^ = 0.86 in the present study sample) with the known FG-associated variant rs11708067. Both *ADCY5* variants are designated GeneHancer and SuperEnhancer variants, and rs72964564 is in an active enhancer state for adipose tissue and is an eQTL associated with ADCY5 expression in pancreatic islets^16^. *ADCY5* and rs72964564 have been previously identified in studies of FG (Supplementary Data 3–4).

Near the *APOB* region a suggestively associated variant at rs478588 (*P* = 2.9 × 10^−9^) has not previously identified (Supplementary Data 4). Variant rs478588 has robust associations with lipid traits^20^ and significant parent-of-origin effects on metabolic traits^21^. Lipid traits have been studied for pleiotropy with glycemic traits but have been inconclusive with respect to *APOB*. Replication was attempted in UK-BioBank (UKBB) with consistent direction of effect and *P* = 0.01, but it should be noted UKBB sample used was not based on WGS data (Supplementary Table 5).

We identified a pair of suggestively FG-associated signals in islet-specific active enhancer regions at the known *SLC30A8* locus. The primary signal is at variant rs35859536 (*P* = 1.0 × 10^−9^), which is an intergenic variant 2.5KB downstream of *SLC30A8*. This variant is highly linked (*r*^2^ > 0.95) to previously identified lead variants rs11558471 and rs3802177 at *SLC30A8*, both of which are in the 3′ UTR. This is a known T2D susceptibility locus and has been identified as associated with triglyceride levels^22^. Our lead variant is also significantly associated with T2D in TOPMed (Supplementary Data 5)^23^. To evaluate potential causal variants (“Methods”) we performed credible set analyses and found rs35859536 has a posterior probability (PP) of 0.48; other variants in the 95% credible set with PP of at least 0.05 were either missense or in the 3′ UTR, are highly linked with this lead variant (*r*^2^ > 0.97), and were significantly associated with FG in previous studies^2,8,24^. Our lead variant, along with other previous lead variants, is in an active enhancer 2 region for islets; rs35859536 is also mapped as an accessible chromatin site in islet of Langerhans given inflammatory-inducing cytokine exposure^25^. Replication of these signals was attempted in the METSIM cohorts, and we observe nominal significance of the primary signal with a consistent direction of effect, while the secondary signal was too low frequency in this cohort to estimate an effect (Supplementary Table 6).

The secondary suggestively FG-associated signal at the *SLC30A8* locus is at variant rs542965166 (*P* = 1.9 × 10^−6^). This intergenic variant is only observed in individuals in the Asian population (Asian EAF = 0.007); this race/ethnicity-specificity is replicated in gnomAD^26^ where the allele is only observed in East Asians at a rare frequency. This secondary, race/ethnicity-specific signal is not highly linked to other variants in the region, which may indicate that this is a distinct, secondary signal and requires further follow-up in an Asian population.

Upstream of the *ROBO1* locus we identified a suggestively novel (to the best of our knowledge) FI-associated rare variant, rs539973028 (*P* = 4.7 × 10^−8^). This locus has previously been studied for *SLIT*-*ROBO* signaling and expression in T2D complication diabetic retinopathy^27^. *ROBO1* has been associated with the glycemia-related traits of BMI and waist-to-hip ratio^28–30^ and is commonly expressed in muscle and skin^31^. This variant is only observed in the African population of TOPMed and gnomAD^26^. It is intergenic and in a weakly transcribed region in islets.

We identified a pair of distinct, suggestively novel (to the best of our knowledge) rare variant signals associated with FI near the *PTPRT* gene (Table 2). The primary signal, rs185250851 (*P* = 2.1 × 10^−8^), is an intronic variant. It is rare in all tested population groups and not observed in Asian individuals, as validated in gnomAD^26^. The secondary signal at variant rs78618809 (*P* = 5.9 × 10^−6^) is in an intergenic region. This variant is within the top 5% of variants with respect to an aPC representing “Distance to TSS/TSE,” a composite measure of individual annotations indicating low variant distance to the endpoints of the intergenic region. This variant is rare overall, but observed frequently (EAF = 0.07) in the African ancestry population. This gene has previously been associated with BMI, which has moderate genetic correlation (previously estimated as *ρ*_g_ = 0.48) with FI^20,21^. The expression of this gene is most commonly associated with variants as eQTLs in pancreas in GTEx^31^. The multi-ethnic TOPMed sample permits the identification of this signal, which requires a sufficiently diverse sample.

### Race/ethnicity-specific analyses associated with fasting glucose and insulin

In race/ethnicity-specific analyses, we observed two not previously identified race/ethnicity-specific rare variant suggestive associations with FG in individuals of the Hispanic/Latinx population (Table 2). The first signal, rs1328056 (*P* = 3.6 × 10^−8^), is an intronic variant in the *HS6ST2* gene, which has been associated with obesity and impaired glucose metabolism in mouse studies^13^. The second signal is an intergenic variant near the *ATPSCKMT* gene, rs13361160 (*P* = 3.1 × 10^−8^) which is associated with eosinophil counts, a measure that has been negatively correlated with FG^14^. We would require further data from individuals from the Hispanic/Latinx population in order to replicate these suggestive signals.

We identified two suggestively novel (to the best of our knowledge) race/ethnicity-specific rare alleles associated with FI. In the European population, rs775018107 (*P* = 4.5 × 10^−8^) at the *LINC00704*/*LINC00705* locus was suggestively associated with FI (Table 2). We also identified a suggestive FI association in the Samoan population cohort at rs117592405 (*P* = 3.3 × 10^−8^); this intronic variant was not replicated in an independent Samoan cohort using imputed genotypes (*N* = 1401, Supplementary Table 7).

### Enrichment of trait-associated variants in chromatin states

We assessed whether our trait-associated variants were found more often than expected in a particular chromatin state using the tool GREGOR (Genomic Regulatory Elements and Gwas Overlap algorithm)^15^ (“Methods”). We observe that fasting glucose-associated variants are found more often in “Active Enhancers”, “Weak Transcription”, and “Genic Enhancer” chromatin states in Islets (*P* < 0.05, Supplementary Table 8). This complements findings from Chen et al.^12^ showing similar enrichment of glycemic trait-associated signals in islet enhancers.

## Discussion

In this paper, we leveraged high-coverage WGS data in large multi-ethnic population-based cohorts to assemble a comprehensive catalog of nucleotide-resolution genomic variation associated with the key diabetes-related quantitative traits FG and FI. Our analysis covered intergenic and intronic regions to a MAC of 20 in single variant analysis and combines base pair variation with tissue-specific epigenomic annotation to illuminate variant-to-function hypotheses in diabetes pathobiology.

A strength of the present analysis is the inclusion of individuals from 15 cohorts, comprised of four major race/ethnicity groups and one population group(African, Asian, European, Hispanic/Latinx, and Samoan, respectively). Some of our reported regions were either mostly or exclusively present in a single race/ethnic group. These include the secondary *SLC30A8* variant rs542965166 only observed in the Asian group, *ROBO1*’s rs539973028 only present in African group, and others. Previous genetic studies of glycemic traits have included samples primarily from individuals of European ancestry, but increasingly a larger degree of African ancestry. The most recent meta-analysis by the MAGIC consortium included ~30% non-European ancestry individuals, demonstrating that a number of trait-associated loci that would have been undetected in samples exclusively of European ancestry^18^. While extending the genetic ancestries studied beyond European populations, the MAGIC results were subject to the limitation of imputation by the 1000 Genomes Project reference panel, so most rare and ancestry-specific variation was still not assessed. In addition, we observe a 20% decrease in average 99% credible set size from the MAGIC results suggesting value of WGS in fine-mapping.

This analysis benefits from the availability of whole-genome sequencing data provided by the TOPMed Program of the NHLBI’s Precision Medicine Initiative^10,32^. Previous studies have been limited by reliance on imputation or minimal sample sizes for data with sequencing paired with glycemic phenotypes. The GoT2D study has performed WGS in a limited sample, contributing to the larger DIAMANTE meta-analysis of summary statistics but relying on imputation for complete genotyping of most samples^33^. The UKBB study includes a large set of primarily European individuals with whole-exome sequencing; however, the sample size with measured fasting glycemic traits is limited as described in the validation study. One of the most expansive efforts, a MAGIC collaboration^8,34^, has performed extensive analyses for glycemic traits, but results rely primary on Exome Chip data and thus have limited coverage of intergenic and intronic regions^6^. Our significant findings replicate previous GWAS findings in terms of gene regions, but we are able to characterize these regions in great detail and report on specific variants which may not previously described in these known regions, such as the secondary *MTNR1B*-associated variant.

In addition to reporting significant and suggestive associations, we provide detailed characterization of each locus in terms of functional annotations, chromatin states, quantitative trait loci, related trait associations and more. The *G6PC2* in particular was described in terms of allelic effects and provided functional characterization of low-frequency signal, demonstrating the glucose-lowering effects of rare alleles and islet-specificity of this locus’s associations. Many of our reported regions lie in enhancer or transcription start site chromatin states, and we particularly see significant enrichment in enhancer states in islets. This agrees with findings of previous GWAS and the expected relevant tissues for glycemic traits. We provide this data and the visualizations for use in future investigation of these loci.

A limitation of this study is our smaller sample size compared to the most recent GWAS. Our significant single variant results are all found near previously identified gene regions. Also, many of our suggestively novel results lack substantiating replication, particularly those which are race/ethnicity specific. We analyzed independent studies with genetic data to investigate associations significant in TOPMed; we were unable to replicate potentially novel signals in these external cohorts. This may be attributable to limitations in the available replication studies’ samples with respect to size, race/ethnicity and imputed versus WGS genotypes. To support the understanding of these signals, we consider a set of tissue-specific chromatin states, an effort that would benefit from further tissue-specific characterization across functional measures. This could also help inform the underlying biological mechanisms of glycemic regulation and its role in diabetes.

This multi-ethnic WGS study provides the foundation for future sequencing-based investigation of glycemic traits. Our results from common and rare variant analysis comprised multiple suggestive hits, including those with exceedingly rare variants that require further investigation, indicating the potential for the identification of novel signals given larger sequencing studies and external validation studies. The value of diverse studies like TOPMed is further evidenced by the specificity of such signals to certain populations and cohorts. This value is also demonstrated by the intronic and intergenic location of many such suggested signals. These signals, in both single variant and rare variant set-based testing, indicated that many associations lie outside gene boundaries and it is important to perform genome-wide single variant testing but also complement gene-centric RV testing with region-based RV testing to fully capture signal. Future TOPMed study phases will permit the continued investigation of these signals empowered by increased sample sizes, with future directions including detailed fine-mapping of signal regions and assessment of glycemic trait heritability. To support future research, all results from this analysis have been made available to the research community through the Type 2 Diabetes Knowledge Portal (Genetic Association Data will be released in January 2021)^17^.

## Methods

### Whole-genome sequencing

Whole-genome sequencing of blood samples for all participants included deep coverage (>30x on average) sequencing from blood samples provided by the NHLBI TOPMed program. Sequencing was performed across six centers (Broad Institute of MIT and Harvard, Northwest Genomics Center, New York Genome Center, Illumina Genomic Services, Macrogen, and Baylor College of Medicine Human Genome Sequencing Center) as previously described^35^. The TOPMed Informatics Research Center at the University of Michigan performed data harmonization and joint variant discovery and genotype calling, requiring DNA sample contamination below 3% and at least 95% of the genome with at least 10x coverage. Freeze 5b was aligned to GRCh38 reads from the 1000 Genomes Project reference sequences^36^. The samples were further processed by a centralized pipeline by the TOPMed Data Coordinating Center at the University of Washington, where further quality control and sample-identity resolution were performed, including sex and relatedness concordance and selection of variants with missingness <5% and QUAL > 127. Variants were also checked via an excess heterozygosity filter (EXHET), which removed the variant if the Hardy-Weinberg disequilibrium *p*-value was <1 × 10^−6^, after accounting for population structure. After processing, Freeze 5b contained 54,508 samples with 438 million single nucleotide variants (SNVs) and 33 million short insertion-deletion variants.

Population structure principal components were calculated across all Freeze 5b TOPMed participants using PC-AiR; a genetic relatedness matrix was calculated across all Freeze 5b TOPMed participants using PC-Relate accounting for population structure. Race/ethnicity was determined by self-report from each study. Self-reported race/ethnicity was used in conjunction with principal component and/or genetic relatedness matrix adjustment to control for both genetic and individually identified ancestry^37^.

### Phenotype harmonization

Phenotype harmonization proceeded following a protocol defined by the TOPMed Diabetes Working Group for participating TOPMed studies. Duplicated individuals were excluded following the TOPMed Diabetes Working Group protocol. Within a study, monozygotic twins were retained and the duplicate to be kept was chosen based on verification of cohort characteristics, including proper cohort sequencing center designation, and then by highest call rate. Across studies, duplicates were selected by removing missing trait data, prioritizing population-based cohorts, and retaining individual records with the longest follow-up period. All study participants provided informed consent and each study was approved by their respective institutional review boards.

Glycemic traits (fasting glucose (FG) and fasting insulin (FI)) were analyzed for individuals who did not have diabetes at the time of glycemic trait measurement. This subset was defined as those not taking anti-diabetes medication, with fasting glucose <7 mmol/l and/or HbA1c < 6.5%. For individuals with multiple blood draws, the earliest exam or most complete exam was used. Age, sex, and BMI covariates were reported at the time of glycemic trait measurement. Fasting was defined to be at least 8 h without food or drink; measurements from blood were converted to plasma values using a 1.13 correction factor^38^. The units for glucose are mmol/l; units for insulin are pmol/l. Fasting insulin was natural log-transformed prior to analysis in order to address non-normality.

### Study sample and power

The present analysis included 23,211 (FI) and 26,807 (FG) individuals from the NHLBI TOPMed program. The cohorts included consist of participants of self-reported African American (FI *n* = 6803; FG *n* = 7174), East Asian (FI *n* = 572; FG *n* = 2217), European (FI *n* = 13,281; FG *n* = 14,513), Hispanic/Latinx (FI *n* = 1641; FG *n* = 1989), and Samoan (FI *n* = 914; FG *n* = 914) race/ethnicity. Our analysis of fasting insulin included 14 cohorts and fasting glucose included 15 cohorts. The sample is predominantly of European race/ethnicity (FI 57.2%; FG 54.1%) and female (FI 66.5%; FG 65.2%); full cohort descriptions are given in Supplementary Tables 2 and 3.

We performed a post hoc power calculation to evaluate the power to detect genetic signal at the genome-wide threshold for statistical significance of 5 × 10^−8^. Given the study sample size, this analysis was powered to detect 0.54–4.21% and 0.57–4.21% percent variation in glycemic trait explained by a genotype in race/ethnicity-specific analyses for FG and FI, respectively. The pooled study including all samples was powered to detect 0.16% and 0.17% percent variation in glycemic trait explained by a genotype for FG and FI, respectively.

### Single-variant analysis

We performed single variant analysis in Freeze 5b of TOPMed using race/ethnicity-specific and pooled approaches. We tested 64,675,008 variants for associations with FG and 58,759,883 with FI in both pooled and race/ethnicity-specific analyses, and restricted analysis to variants with minor allele count >= 20. We used linear mixed effects models and adjusted for age, age squared, sex, body mass index, study-race/ethnicity, with heterogeneous variance permitted across study-race/ethnicity groups and empirical kinship for relatedness and population structure. Models were fit using GENetic Estimation and Inference in Structured samples (GENESIS)^39^ in the Analysis Commons cloud-computing platform^40^. *P*-Values reported are for a two-sided Wald test from the mixed model. Fasting glucose and natural log-transformed fasting insulin were used as outcomes in separate models. We define the standard genome-wide threshold for statistical significance as 1 × 10^−9^. We also report variants with *P* < 5 × 10^−8^ as suggestively associated to provide context for regions of interest for future, higher-powered studies.

Stepwise conditional analysis was performed at each identified locus, defined to be a 500 kb region centered on the most significant variant, in order to identify distinct signals. This analysis proceeded by first including the most significant variant as a covariate, and repeating until no variants were associated with the phenotype with *p*-value <1 × 10^−5^. For each distinct signal, a final model was run conditioning on the set of other distinct signals; we report these potentially distinct signals.

Towards fine-mapping the identified loci, we generated 95% credible sets to investigate likely causal variants (LocalZoom). For each locus, we calculated Bayes factors for all variants from their single variant *p*-value; *p*-values were taken from conditional analyses on all other identified variants at the locus where multiple distinct signals were identified in the stepwise conditional analysis. We calculated posterior probability of association (PP) of each variant as the proportion contributed to the summation of all BFs in the region. The variants were sorted by descending PP, indicating decreasing probability that the variant is truly associated with the glycemic trait. The 95% credible set was constructed by including variants, starting with the highest PPA, until their cumulative PPA exceeded 0.95. 99% credible sets were similarly constructed for association signals from the pooled analysis only.

### Rare variant analysis

We performed gene-based and genetic region aggregate testing to identify sets of rare variants associated with fasting glucose and log-transformed fasting insulin. We first fit a heteroscedastic linear mixed model for fasting glucose and log-transformed fasting insulin. Both traits were adjusted for age, age^2^, sex, body mass index (BMI), study-race/ethnicity group indicators, and ten population structure principal components. A variance component was included for the empirically derived sparse kinship matrix and residual variances were permitted to be different for study-race/ethnicity groups to account for family relatedness, population structure, and study-race/ethnicity differences.

The heteroscedastic linear mixed model was used to perform variant set analyses for rare variants with MAF < 1%. Sets were defined by genetic regions and gene-centric categories. Genetic regions allowed the complete analysis of the genome, particularly non-coding regions that have not been previously captured by arrays. The regional analysis used 2 kb sliding windows to scan the genome with 1 kb skip length. The gene-centric analysis examined all protein-coding genes in Ensembl using functionally determined masks to aggregate variants together by coding and non-coding annotations. Coding annotations were used to define three SNV filters categorized by GENCODE based on consequence: (a) putative loss of function (stop gain, stop loss, splicing), (b) missense, and (c) synonymous variants. Leveraging the whole-genome sequencing, we used non-coding annotations to test sets of variants that are not protein coding. We constructed masks (d) characterized as promoters given they were within +/− 3 kb of a transcription start site with CAGE signal overlay, or (e) characterized as enhancers given they were identified by GeneHancer with CAGE signal overlay.

The burden test and SKAT were used for testing the association of the rare variant sets and FG and FI. In these approaches, a weight based on the MAF can be used to upweight rarer variants. We considered two common weighting schemes based on ${w}_{j}={{{{{{\rm{Beta}}}}}}}({{{{{{{\rm{MAF}}}}}}}}_{j};{a}_{1},{a}_{2})$, where ${a}_{1}=1$ and ${a}_{2}=25$ or ${a}_{1}=1$ and ${a}_{2}=1$.

Statistical significance was defined for each glycemic trait, separately for gene-centric and genetic region analysis. For gene-centric analysis, a threshold was defined by a Bonferroni-corrected significance threshold of $\alpha \approx 0.05/(120,000)=4\times {10}^{-7}$, correcting for all five masks and all protein-coding genes when considering the minimum *p*-value across the burden and SKAT tests (Supplementary Table 9). The threshold for the genetic region analysis was determined given the total number of 2 kb sliding windows tested, yielding a Bonferroni-corrected threshold of $\alpha \approx 0.05/(2.68\times {10}^{6})=1.86\times {10}^{-8}$. We report sets that include variant(s) with effective minor allele count greater than five and that are not exclusively composed of singletons; complete results based on the significance threshold are provided in Supplementary Data 6–9.

### Haplotype analysis

We performed haplotype analysis for variants associated with fasting glucose. This analysis considered a set of 18,071 unrelated individuals, identified by PC-AiR^41^ by the TOPMed Program with a threshold of third-degree relatives. We performed regression of fasting glucose on haplotype using a two-step EM algorithm on the unphased genotypes, as implemented in the haplo.stats R package^42^. The posterior probabilities of haplotypes were computed for variants in the *G6PC2* gene; the variants were included based on the variants included in a previous *G6PC2* haplotype analysis, variants driving the *G6PC2* missense set signal, and distinct *G6PC2* signals from the single variant analysis. The association was adjusted for age, age^2^, sex, body mass index, study-race/ethnicity, and ten population structure principal components.

### Annotation

In order to characterize the functional impact of associated variants, we assembled functional annotations from multiple publicly available databases. We considered annotations from the Diabetes Epigenome Atlas, FAVOR, InsPIRE, and GTEx projects. From the Diabetes Epigenome Atlas, we obtained chromatin states from four tissues relevant to glycemic traits: adipose, islet, liver, and muscle. These were available from two experiments, Parker lab ChromHMM 13-state model under accession TSTSR679993 & AMP-T2D ChromHMM 18-state model under accession TSTSR043890. We also report annotation PCs from the FAVOR database^43^, which are summaries calculated as the first principal component of individual functional annotations across functional categories including conservation, epigenetics, local nucleotide diversity, mutation density, protein function, proximity to TSS/TSE, proximity to coding, and transcription factor binding. The individual annotations contributing to the aPCs are previously described^19^. Annotation PCs are calculated at the variant level and reported as PHRED-scaled scores derived from the first PC from the category’s PCA, providing the interpretation that variants with scores >10 are in the top 10% of category across all TOPMed variants. We obtained pancreatic islet-specific signals from the InsPIRE consortium and tissue-specific signals from the GTEx project (Version 8) to assess colocalization with gene expression at signal variants and those highly linked to signal variants via look-up. We reported eQTLs in the following tissues, reported for their importance in glycemic phenotypes: adipose subcutaneous, adipose visceral, muscle skeletal, and pancreas.

### Replication

We sought to replicate our findings in the METSIM study^44^, using data from 10,058 individuals with fasting glucose, fasting insulin, and TOPMed-imputed genotypes. EMMAX was used to test for associations with fasting glucose or log-transformed fasting insulin at the variants reported in Table 1 with age, age^2^, and BMI as covariates and kinship; sex was not included as a covariate as the study is all males.

We additionally performed replication analysis in a sample from the UK Biobank. A sample of 12,854 European ancestry individuals from the UK Biobank with glucose was selected from all individuals with glucose measurement, excluding individuals with diabetes (Data-field 2443), on diabetes medication (Data-field 6177/6153), or with fasting time <8 h (Data-field 74). Glucose values were taken from variable 30740. The model included age (Data-field 21022), age^2^, sex (Data-field 31), BMI (Data-field 21001), and ten population structure PCs. Association models were run using Scalable and Accurate Implementation of GEneralized mixed model (SAIGE)^45^ to analyze UKBB phenotype data and the imputed chip genetic data.

This research has been conducted using the UK Biobank Resource under Application Number 42614.

We also performed replication analysis of the Samoan-specific association of rs117592405 with fasting insulin in a cohort of 1401 Samoans without WGS from the Samoan Study. rs117592405 was imputed using a Samoan-specific reference panel that was developed from the WGS of 1284 Samoans as part of TOPMed. R version 3.6.0 was used to replicate the association with fasting insulin in individuals without a previous diabetes diagnosis or diabetes medication use. Age, age^2^, BMI, and sex were included in the model.

### Enrichment

The tool GREGOR was used to assess if our trait-associated variants in Table 1 were significantly enriched in a particular chromatin state annotation. Using computed LD from the 1000-genomes reference panel and the 18-state chromatin model described in the text and shown in Fig. 1, we obtained an expected number of variants to lie within each chromatin state. This was compared to the observed number of variants in each chromatin state to generate a *P*-value. Any *P*-values <0.05 are reported in the text and Supplementary Table 8.

### Reporting summary

Further information on research design is available in the Nature Research Reporting Summary linked to this article.

## Supplementary information

Supplementary Material

Description of Additional Supplementary Files

Supplementary Data 1-9

Reporting Summary

## Data Availability

The summary results generated during this study are available at the AMP-T2D Portal, http://t2d.hugeamp.org/. Fasting Insulin: https://t2d.hugeamp.org/dinspector.html?dataset=TOPMed_frz5b_pooled_FI_WGS. Fasting Glucose: https://t2d.hugeamp.org/dinspector.html?dataset=TOPMed_frz5b_pooled_FG_WGS. Accession codes for genotype and phenotype files by cohort may be found in Supplementary Table 1.

## Code availability

This study did not rely on custom code or mathematical algorithms.
