# Supplementary material for: Whole genome sequence association analysis of fasting glucose and fasting insulin levels in diverse cohorts from the NHLBI TOPMed program
Source: Commun Biol. 2022 Jul 28;5:756. doi: 10.1038/s42003-022-03702-4 (PMC9334637; doi:10.1038/s42003-022-03702-4)
Supplement: Supplementary file 1 — Supplementary Material [file 42003_2022_3702_MOESM1_ESM.pdf]

# SUPPLEMENTARY INFORMATION

## CONSORTIA AUTHORS AFFILIATIONS

### NHLBI Trans-Omics for Precision Medicine (TOPMed) Consortium Banner Author’s Affiliations:

[1]New York Genome Center, New York, New York, 10013, US, [2]University of Michigan, Ann Arbor, Michigan, 48109, US, [3]Broad Institute, Cambridge, Massachusetts, 2142, US, [4]Brigham & Women's Hospital, Cedars Sinai, Boston, Massachusetts, 2114, US, [5]Children's Hospital of Philadelphia, University of Pennsylvania, Philadelphia, Pennsylvania, 19104, US, [6]Emory University, Atlanta, Georgia, 30322, US, [7]University of Maryland, Baltimore, Maryland, 21201, US, [8]University of Washington, Seattle, Washington, 98195, US, [9]University of Mississippi, Jackson, Mississippi, 38677, US, [10]National Institutes of Health, Bethesda, Maryland, 20892, US, [11]Johns Hopkins University, Baltimore, Maryland, 21218, US, [12]University of Kentucky, Lexington, Kentucky, 40506, US, [13]Duke University, Durham, North Carolina, 27708, US, [14]University of Alabama, Birmingham, Alabama, 35487, US, [15]Stanford University, Stanford, California, 94305, US, [16]University of Wisconsin Milwaukee, Milwaukee, Wisconsin, 53211, US, [17]Cleveland Clinic, Cleveland, Ohio, 44195, US, [18]University of Colorado Anschutz Medical Campus, Aurora, Colorado, 80045, US, [19]Columbia University, New York, New York, 10027, US, [20]LTRC, The Emmes Corporation, Rockville, Maryland, 20850, US, [21]Quantitative Health Sciences, Cleveland Clinic, Cleveland, Ohio, 44195, US, [22]Medicine, Johns Hopkins University, Baltimore, Maryland, 21218, US, [23]National Heart, Lung, and Blood Institute, National Institutes of Health, Bethesda, Maryland, 20892, US, [24]Boston University School of Medicine, Boston University, Massachusetts General Hospital, Boston, Massachusetts, 2114, US, [25]University of Pittsburgh, Pittsburgh, Pennsylvania, 15260, US, [26]Fundação de Hematologia e Hemoterapia de Pernambuco - Hemope, Recife, 52011-000, BR, [27]Cardiovascular Health Research Unit, Department of Medicine, University of Washington, Seattle, Washington, 98195, US, [28]Human Genetics, University of Texas Rio Grande Valley School of Medicine, Brownsville, Texas, 78520, US, [29]University of Texas Health at Houston, Houston, Texas, 77225, US, [30]Department of Biochemistry, Wake Forest Baptist Health, Winston-Salem, North Carolina, 27157, US, [31]National Jewish Health, National Jewish Health, Denver, Colorado, 80206, US, [32]Medical College of Wisconsin, Milwaukee, Wisconsin, 53226, US, [33]University of California, San Francisco, San Francisco, California, 94143, US, [34]Biomedical Data Science, Stanford University, Stanford, California, 94305, US, [35]Biostatistics, University of Washington, Seattle, Washington, 98195, US, [36]Brigham and Women's Hospital, Brigham & Women's Hospital, Boston, Massachusetts, 2115, US, [37]University of Colorado at Denver, Denver, Colorado, 80204, US, [38]Brigham & Women's Hospital, Boston, Massachusetts, 2115, US, [39]Washington State University, Pullman, Washington, 99164, US, [40]University of California, Los Angeles, Los Angeles, California, 90095, US, [41]Medicine, Brigham & Women's Hospital, Boston, Massachusetts, 2115, US, [42]National Taiwan University, Taipei, 10617, TW, [43]Division of Preventive Medicine, Brigham & Women's Hospital, Boston, Massachusetts, 2215, US, [44]University of Virginia,

Charlottesville, Virginia, 22903, US, [45]Lundquist Institute, Torrance, California, 90502, US, [46]National Taiwan University Hospital, National Taiwan University, Taipei, 10617, TW, [47]Cleveland Clinic, Cleveland Clinic, Cleveland, Ohio, 44195, US, [48]National Health Research Institute Taiwan, Miaoli County, 350, TW, [49]Metabolomics Platform, Broad Institute, Cambridge, Massachusetts, 2142, US, [50]Immunity and Immunology, Cleveland Clinic, Cleveland, Ohio, 44195, US, [51]University of Vermont, Burlington, Vermont, 5405, US, [52]Medicine, University of Mississippi, Jackson, Mississippi, 38677, US, [53]National Jewish Health, Denver, Colorado, 80206, US, [54]Biostatistics, Boston University, Boston, Massachusetts, 2115, US, [55]University of Texas Rio Grande Valley School of Medicine, Brownsville, Texas, 78520, US, [56]Vitalant Research Institute, San Francisco, California, 94118, US, [57]University of Illinois at Chicago, Chicago, Illinois, 60607, US, [58]University of Chicago, Chicago, Illinois, 60637, US, [59]Health Sciences Research, Mayo Clinic, Rochester, Minnesota, 55905, US, [60]Department of Medicine, Cardiovascular Division, Washington University in St Louis, St. Louis, Missouri, 63110, US, [61]Vanderbilt University, Nashville, Tennessee, 37235, US, [62]University of Cincinnati, Cincinnati, Ohio, 45220, US, [63]The Samoan Obesity, Lifestyle and Genetic Adaptations Study (OLaGA) Group, [64]University of North Carolina, Chapel Hill, North Carolina, 27599, US, [65]University of Texas Rio Grande Valley School of Medicine, Edinburg, Texas, 78539, US, [66]Washington University in St Louis, St Louis, Missouri, 63130, US, [67]Brown University, Providence, Rhode Island, 2912, US, [68]Channing Division of Network Medicine, Harvard University, Cambridge, Massachusetts, 2138, US, [69]Massachusetts General Hospital, Boston, Massachusetts, 2114, US, [70]Center for Genes, Environment and Health, National Jewish Health, Denver, Colorado, 80206, US, [71]Epidemiology, University of North Carolina, Chapel Hill, North Carolina, 27599, US, [72]Fred Hutchinson Cancer Research Center, Seattle, Washington, 98109, US, [73]Icahn School of Medicine at Mount Sinai, New York, New York, 10029, US, [74]Medicine, Indiana University, Indianapolis, Indiana, 46202, US, [75]Beth Israel Deaconess Medical Center, Boston, Massachusetts, 2215, US, [76]Baylor College of Medicine Human Genome Sequencing Center, Houston, Texas, 77030, US, [77]Yale University, New Haven, Connecticut, 6520, US, [78]University of Texas Rio Grande Valley School of Medicine, San Antonio, Texas, 78229, US, [79]Department of Chronic Disease Epidemiology, Yale University, New Haven, Connecticut, 6520, US, [80]Tulane University, New Orleans, Louisiana, 70118, US, [81]University of California, San Francisco, Montreal, H3A 0G4, CA, [82]Wake Forest Baptist Health, Winston-Salem, North Carolina, 27157, US, [83]Channing Division of Network Medicine, Brigham & Women's Hospital, Boston, Massachusetts, 2115, US, [84]University of Iowa, Iowa City, Iowa, 52242, US, [85]Institute of Population Health Sciences, NHRI, National Health Research Institute Taiwan, Miaoli County, 350, TW, [86]Tri-Service General Hospital National Defense Medical Center, TW, [87]Blood Works Northwest, Seattle, Washington, 98104, US, [88]Taichung Veterans General Hospital Taiwan, Taichung City, 407, TW, [89]Internal Medicine, DIvision of Endocrinology, Diabetes and Metabolism, Ohio State University Wexner Medical Center, Columbus, Ohio, 43210, US, [90]Blood Works Northwest, University of Washington, Seattle, Washington, 98104, US, [91]Biostatistics, University of Michigan, Ann Arbor, Michigan, 48109, US, [92]Albert Einstein College of Medicine, New York, New York, 10461, US, [93]Harvard University, Cambridge, Massachusetts, 2138, US, [94]Public Health Sciences, Loyola University, Maywood, Illinois, 60153, US, [95]Biostats, Harvard School of Public

Health, Boston, Massachusetts, 2115, US, [96]Boston University, Boston, Massachusetts, 2215, US, [97]Harvard School of Public Health, Boston, Massachusetts, 2115, US, [98]Epidemiology and Medicine, Brown University, Providence, Rhode Island, 2912, US, [99]Cardiology, Duke University, Durham, North Carolina, 27708, US, [100]Cardiovascular Institute, Stanford University, Stanford, California, 94305, US, [101]The Charles Bronfman Institute for Personalized Medicine, Icahn School of Medicine at Mount Sinai, New York, New York, 10029, US, [102]George Washington University, Washington, District of Columbia, 20052, US, [103]University of Arizona, Tucson, Arizona, 85721, US, [104]Genes and Human Disease, Oklahoma Medical Research Foundation, Oklahoma City, Oklahoma, 73104, US, [105]Ministry of Health, Government of Samoa, Apia, WS, [106]Broad Institute, Harvard University, Massachusetts General Hospital, Boston, Massachusetts, 2114, US, [107]Howard University, Washington, District of Columbia, 20059, US, [108]University of Maryland, Baltimore, Maryland, 21201, US, [109]University at Buffalo, Buffalo, New York, 14260, US, [110]Stanford Cardiovascular Institute, Stanford University, Stanford, California, 94305, US, [111]Biochemistry, Wake Forest Baptist Health, Winston-Salem, North Carolina, 27157, US, [112]University of Minnesota, Minneapolis, Minnesota, 55455, US, [113]Fred Hutchinson Cancer Research Center, University of Washington, Seattle, Washington, 98195, US, [114]Cardiology/Medicine, Johns Hopkins University, Baltimore, Maryland, 21218, US, [115]Medicine, University of Colorado at Denver, Denver, Colorado, 80204, US, [116]University of Colorado at Denver, Denver, Colorado, 80045, US, [117]Genetics, University of North Carolina, Chapel Hill, North Carolina, 27599, US, [118]Northwestern University, Chicago, Illinois, 60208, US, [119]Fred Hutchinson Cancer Research Center, University of Washington, Seattle, Washington, 98109, US, [120]Lutia I Puava Ae Mapu I Fagalele, Apia, WS, [121]Medicine, Pharmacology, Biomedicla Informatics, Vanderbilt University, Nashville, Tennessee, 37235, US, [122]Faculdade de Medicina, Universidade de Sao Paulo, Sao Paulo, 1310000, BR, [123]TGPS, Lundquist Institute, Torrance, California, 90502, US, [124]Division of Hematology/Oncology, Broad Institute, Harvard University, Cambridge, Massachusetts, 2142, US, [125]Genetics, Harvard Medical School, Boston, Massachusetts, 2115, US, [126]Harvard Medical School, Boston, Massachusetts, 2115, US, [127]Pediatrics, Baylor College of Medicine, Houston, Texas, 77030, US, [128]Human Genetics, Emory University, Atlanta, Georgia, 30322, US, [129]Medicine/Cardiology, Vanderbilt University, Nashville, Tennessee, 37235, US, [130]UMass Memorial Medical Center, Worcester, Massachusetts, 1655, US, [131]Epidemiology, University of Washington, Seattle, Washington, 98195, US, [132]Biostatistical Sciences, Wake Forest Baptist Health, Winston-Salem, North Carolina, 27157, US, [133]Genetics, Stanford University, Stanford, California, 94305, US, [134]Institute for Translational Genomics and Populations Sciences, Lundquist Institute, Torrance, California, 90502, US, [135]University of Washington, Department of Genome Sciences, University of Washington, Seattle, Washington, 98195, US, [136]Genetics, University of Pennsylvania, Philadelphia, Pennsylvania, 19104, US, [137]Biostatistics, University of Alabama, Birmingham, Alabama, 35487, US, [138]Department of Biostatistics, University of Washington, Seattle, Washington, 98195, US, [139]Pathology & Laboratory Medicine, University of Vermont, Burlington, Vermont, 5405, US, [140]USC Methylation Characterization Center, University of Southern California, University of Southern California, California, 90033, US, [141]University of Colorado at Boulder, University of Minnesota, Boulder, Colorado, 80309, US, [142]Brigham & Women's Hospital,

Partners.org, Boston, Massachusetts, 2115, US, [143]Epidemiology, Indiana University, Indianapolis, Indiana, 46202, US, [144]Internal Medicine, University of Michigan, Ann Arbor, Michigan, 48109, US, [145]Henry Ford Health System, Detroit, Michigan, 48202, US, [146]Cardiology, Beth Israel Deaconess Medical Center, Boston, Massachusetts, 2215, US, [147]Medicine, University of Pittsburgh, Pittsburgh, Pennsylvania, 15260, US, [148]Department of Epidemiology, University of Michigan, Ann Arbor, Michigan, 48109, US, [149]Department of Population and Quantitative Health Sciences, Case Western Reserve University, Cleveland, Ohio, 44106, US

# Supplementary Acknowledgements

## TOPMed Omics Support

| TOPMed Accession # | TOPMed Project | Parent Study | TOPMed Phase | Omics Center | Omics Support |
|---|---|---|---|---|---|
| phs001218 | AA_CAC | GeneSTAR AA_CAC | 2 | Broad Genomics[a] | HHSN268201500014C |
| phs001345 | AA_CAC | GENOA AA_CAC | 2 | Broad Genomics[a] | HHSN268201500014C |
| phs001416 | AA_CAC | MESA AA_CAC | 2 | Broad Genomics[a] | HHSN268201500014C |
| phs001211 | AFGen | ARIC AFGen | 1 | Broad Genomics[a] | 3R01HL092577-06S1 |
| phs000974 | AFGen | FHS AFGen | 1 | Broad Genomics[a] | 3R01HL092577-06S1 |
| phs000956 | Amish | Amish | 1 | Broad Genomics[a] | 3R01HL121007-01S1 |
| phs000954 | CFS | CFS | 1 | NWGC[b] | 3R01HL098433-05S1 |
| phs000954 | CFS | CFS | 3.5 | NWGC[b] | HHSN268201600032I |
| phs001368 | CHS | CHS | 3 | Baylor[c] | HHSN268201600033I |
| phs000974 | FHS | FHS | 1 | Broad Genomics[a] | 3U54HG003067-12S2 |
| phs001218 | GeneSTAR | GeneSTAR | legacy | Illumina | R01HL112064 |
| phs001218 | GeneSTAR | GeneSTAR | 2 | Psomagen | 3R01HL112064-04S1 |
| phs001217 | GenSalt | GenSalt | 2 | Baylor[c] | HHSN268201500015C |
| phs001359 | GOLDN | GOLDN | 2 | NWGC[b] | 3R01HL104135-04S1 |
| phs001345 | HyperGEN_ GENOA | GENOA | 2 | NWGC[b] | 3R01HL055673-18S1 |
| phs001293 | HyperGEN_ GENOA | HyperGEN | 2 | NWGC[b] | 3R01HL055673-18S1 |
| phs000964 | JHS | JHS | 1 | NWGC[b] | HHSN268201100037C |
| phs001416 | MESA | MESA | 2 | Broad Genomics[a] | 3U54HG003067-13S1 |
| phs001215 | SAFS | SAFS | 1 | Illumina | 3R01HL113323-03S1 |
| phs001215 | SAFS | SAFS | legacy | Illumina | R01HL113322 |
| phs000972 | Samoan | Samoan | 1 | NWGC[b] | HHSN268201100037C |
| phs000972 | Samoan | Samoan | 2 | NYGC[d] | HHSN268201500016C |
| phs001211 | VTE | ARIC | 2 | Baylor[c] | 3U54HG003273-12S2, HHSN268201500015C |
| phs001368 | VTE | CHS VTE | 2 | Baylor[c] | 3U54HG003273-12S2, HHSN268201500015C |
| phs001237 | WHI | WHI | 2 | Broad Genomics[a] | HHSN268201500014C |

[a]Broad Institute Genomics Platform, [b]Northwest Genomics Center, [c]Baylor College of Medicine Human Genome Sequencing Center, [d]New York Genome Center

# Supplementary Figures

**Supplementary Figure 1.** Manhattan and QQ plots for pooled single variant analysis with fasting glucose and fasting insulin in TOPMed. The Manhattan plots show the $-\log_{10}$ *P-values* for the variants across the chromosomal positions with a threshold of $5\times10^{-8}$ plotted in red; the QQ-plots show the observed versus expected $-\log_{10}$ *P-values*. The plots are stratified by common (MAF>=0.05) and rare (MAF<0.05) variants for all variants with MAC>=20.

a. Fasting Glucose, MAF>=0.05

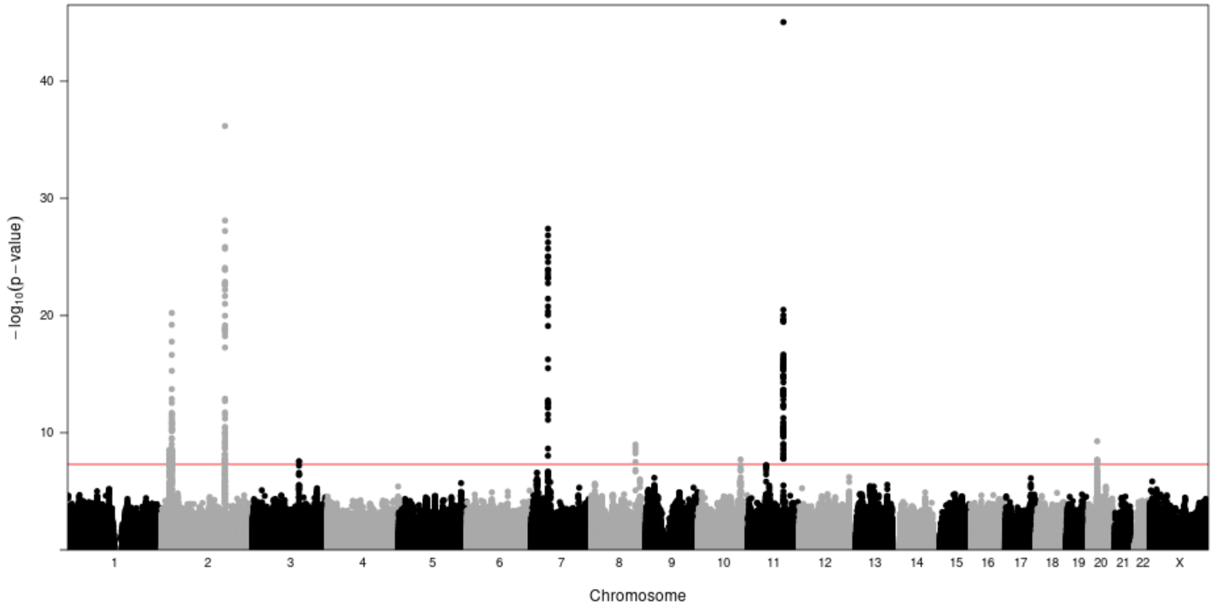


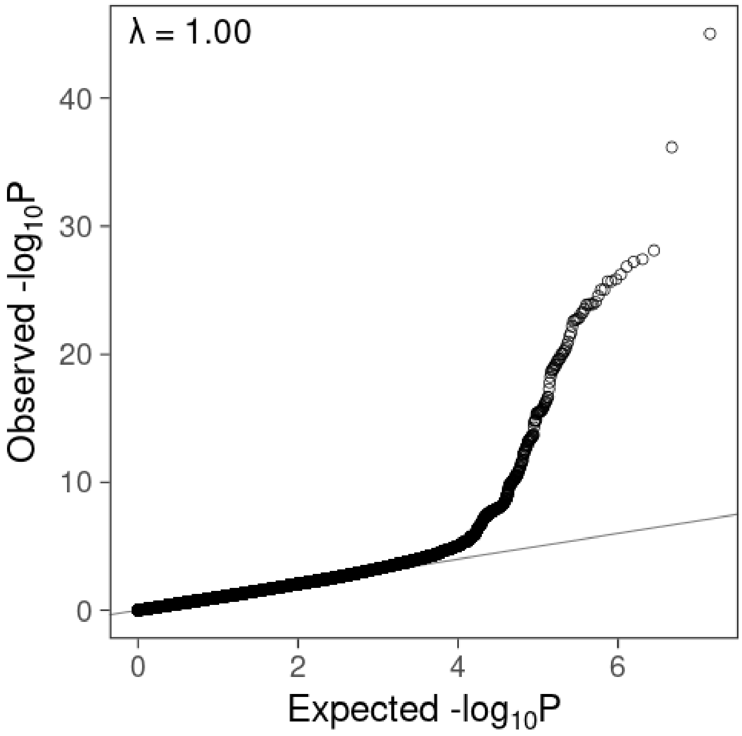

b. Fasting Glucose, MAF<0.05

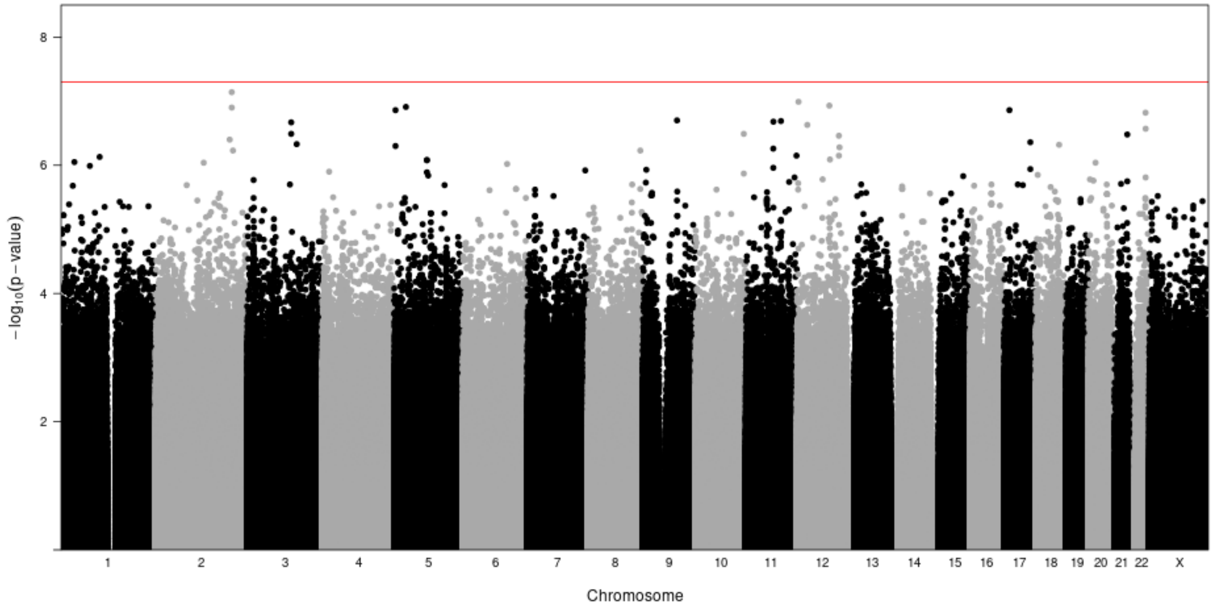


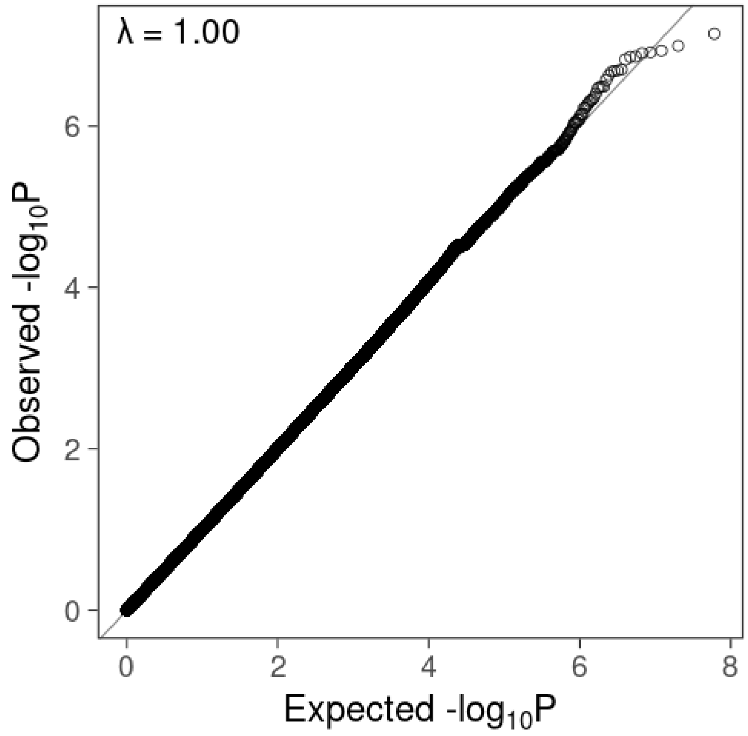

c. Fasting Insulin, MAF>=0.05

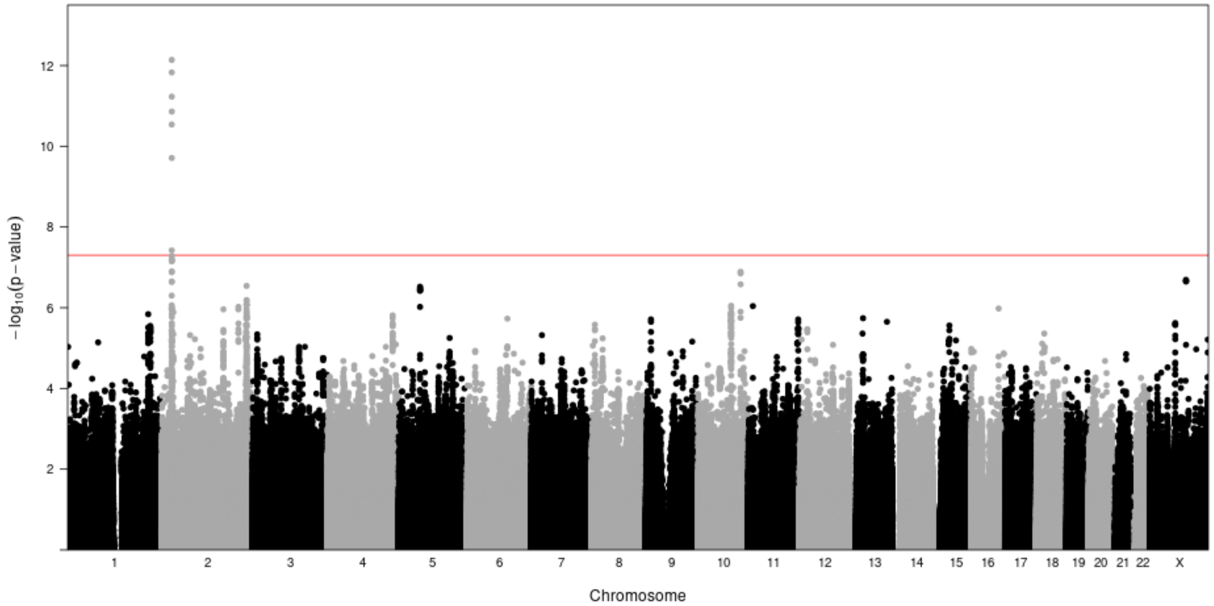


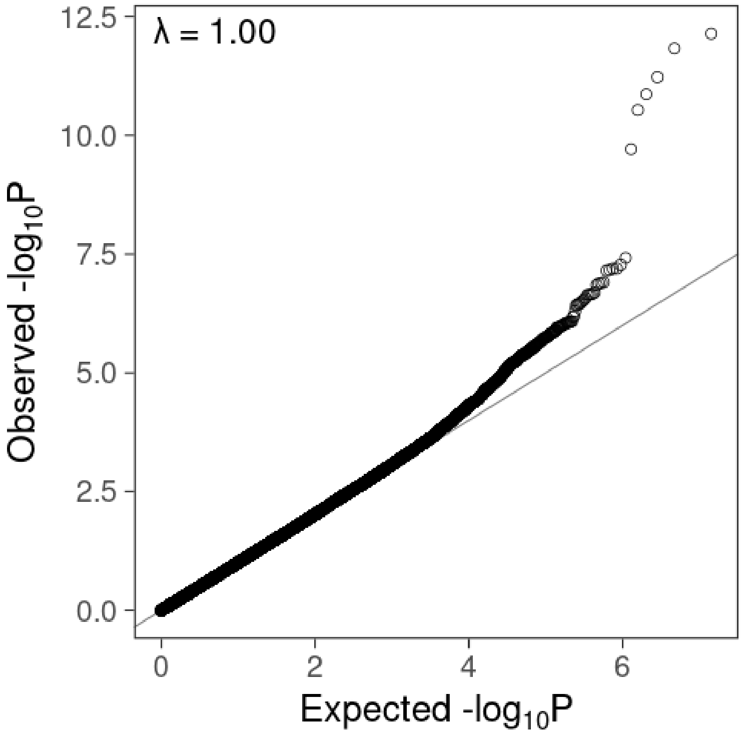

d. Fasting Insulin, MAF<0.05

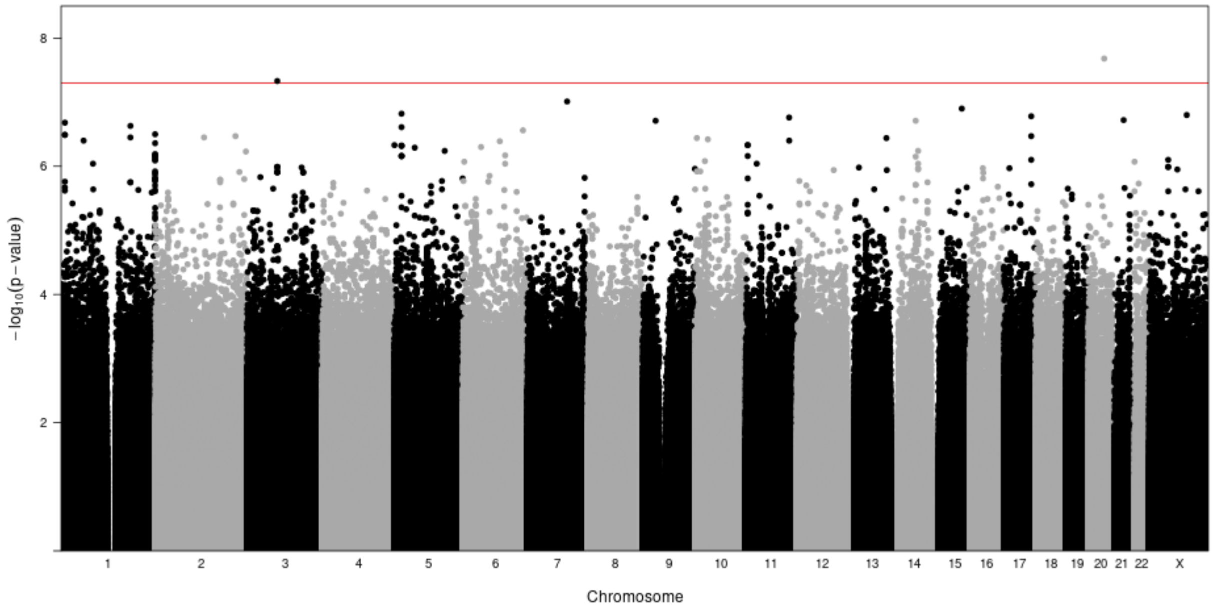


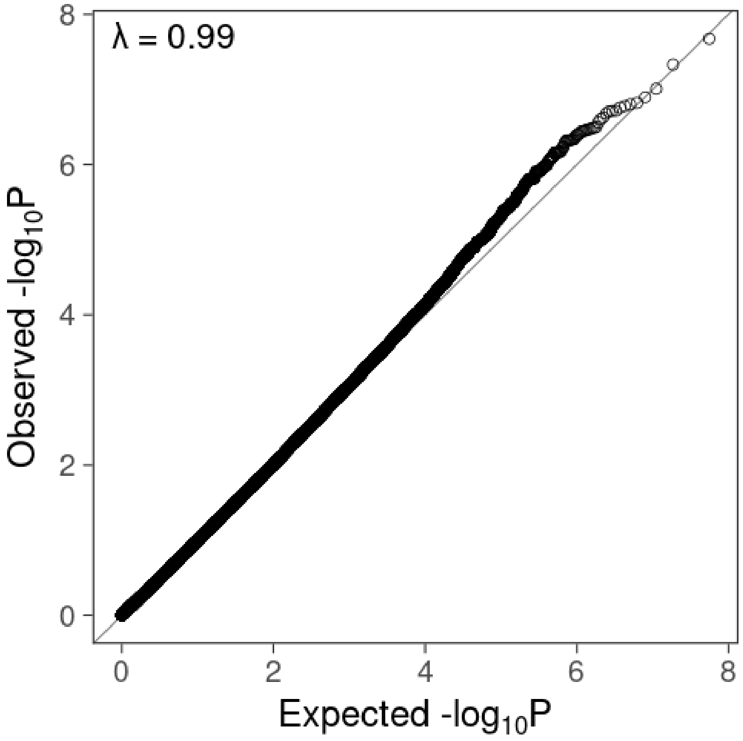

**Supplementary Figure 2.** Regional locus plots of the twelve loci significantly or suggestively associated in pooled analysis with stepwise conditional analysis. The loci associated with FG (a-b. *MTNR1B,* b. *G6PC2,* c. *GCK,* d. *GCKR,* e. *FOX2A,* f. *SLC30A8,* g. *APOB,* h. *TCF7L2,* i. *ADCY5*) and FI (j. *GCKR,* k. *PTPRT,* l. *ROBO1*). The linkage disequilibrium is given with respect to the index variant and was calculated in the TOPMed analysis sample. Plots denoted by rsID and MarkerID (Chromosome:Position(Hg38):Reference-Allele:Alternative-Allele).

a. *MTNR1B*, rs10830963, 11:92975544:C:G

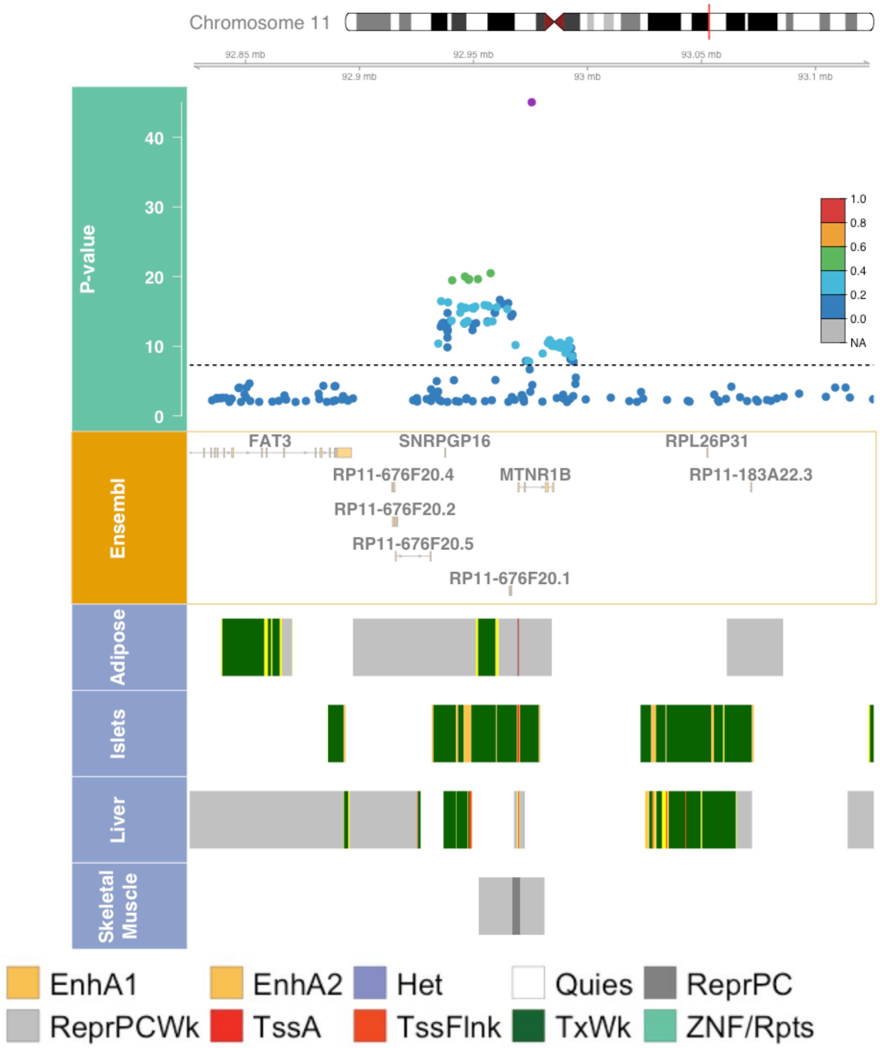

b. *MTNR1B*, rs73560545, 11:92884161:G:A

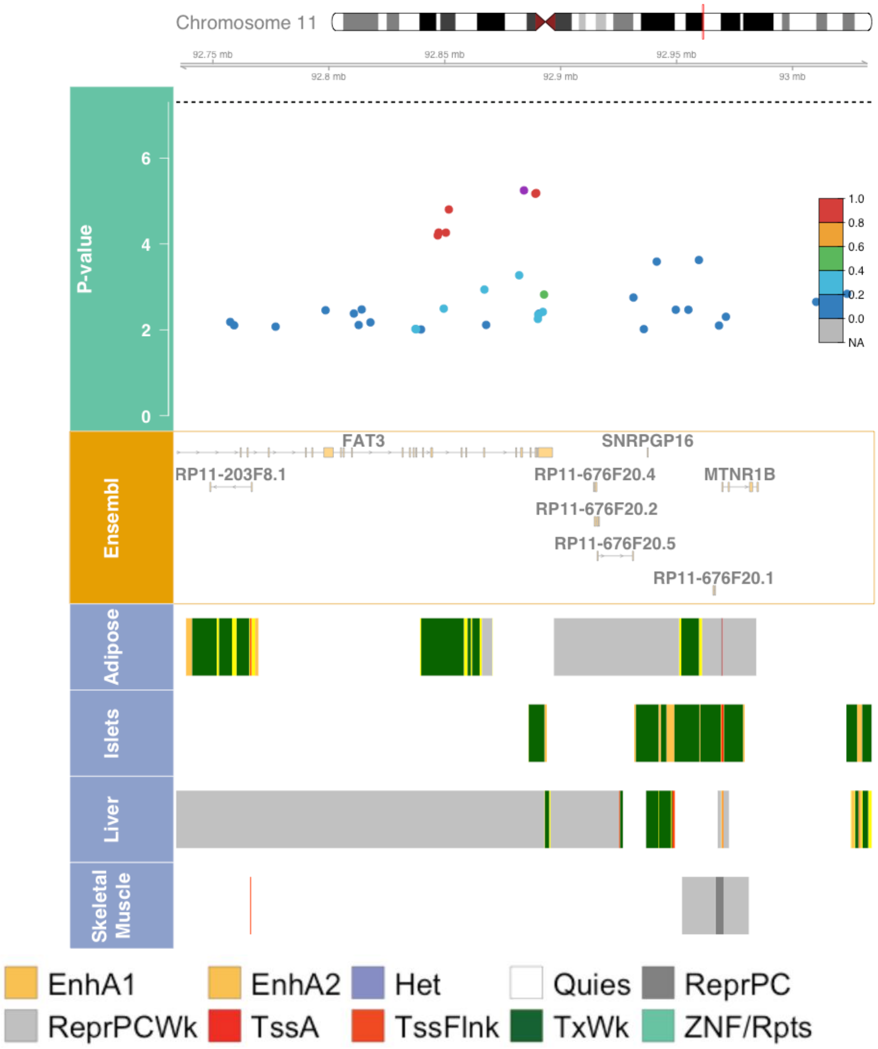

c. *G6PC2*, rs560887, 2:168906638:T:C

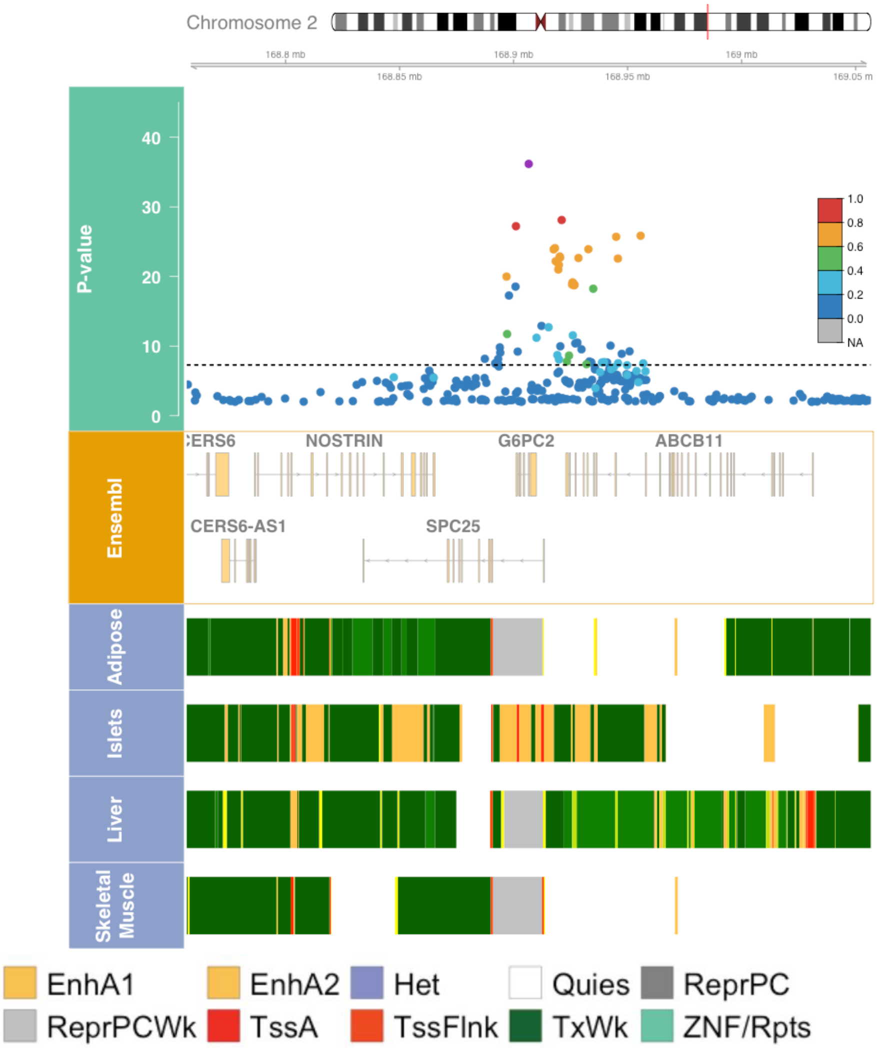

d. *G6PC2*, rs540524, 2:168900420:A:G

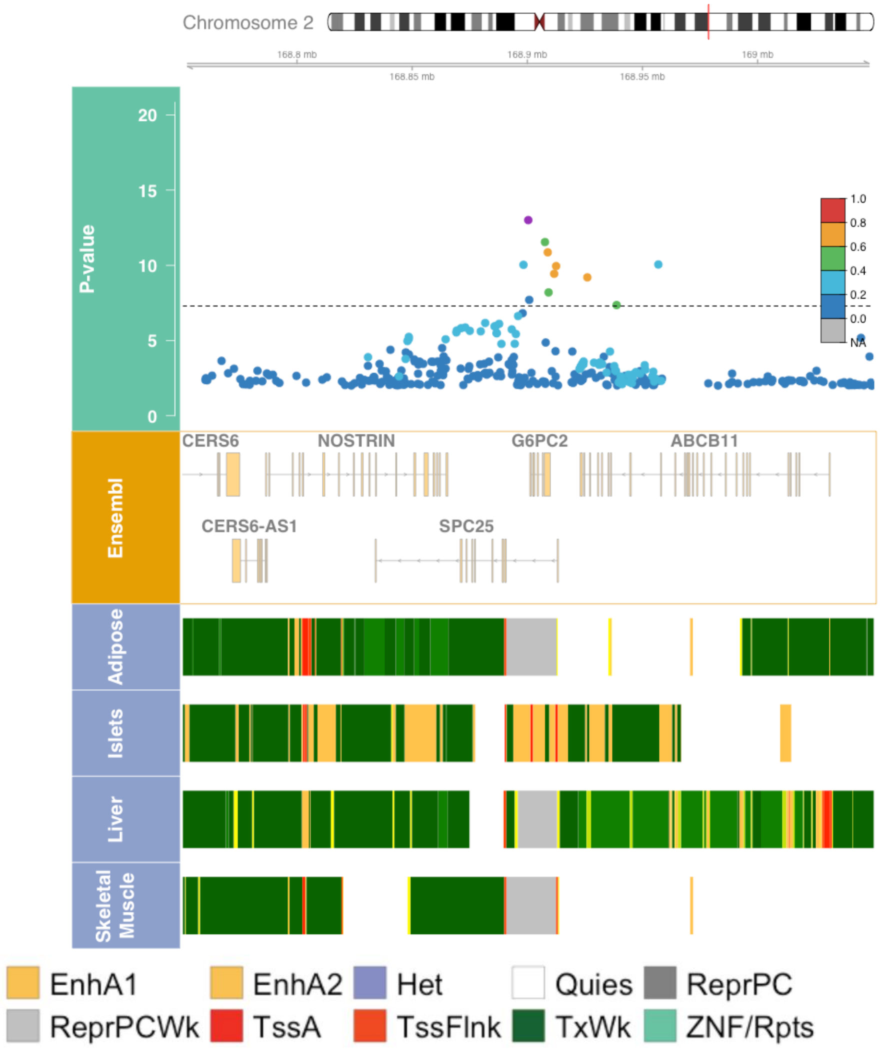

e. *G6PC2*, rs2232326, 2:168907981:T:C

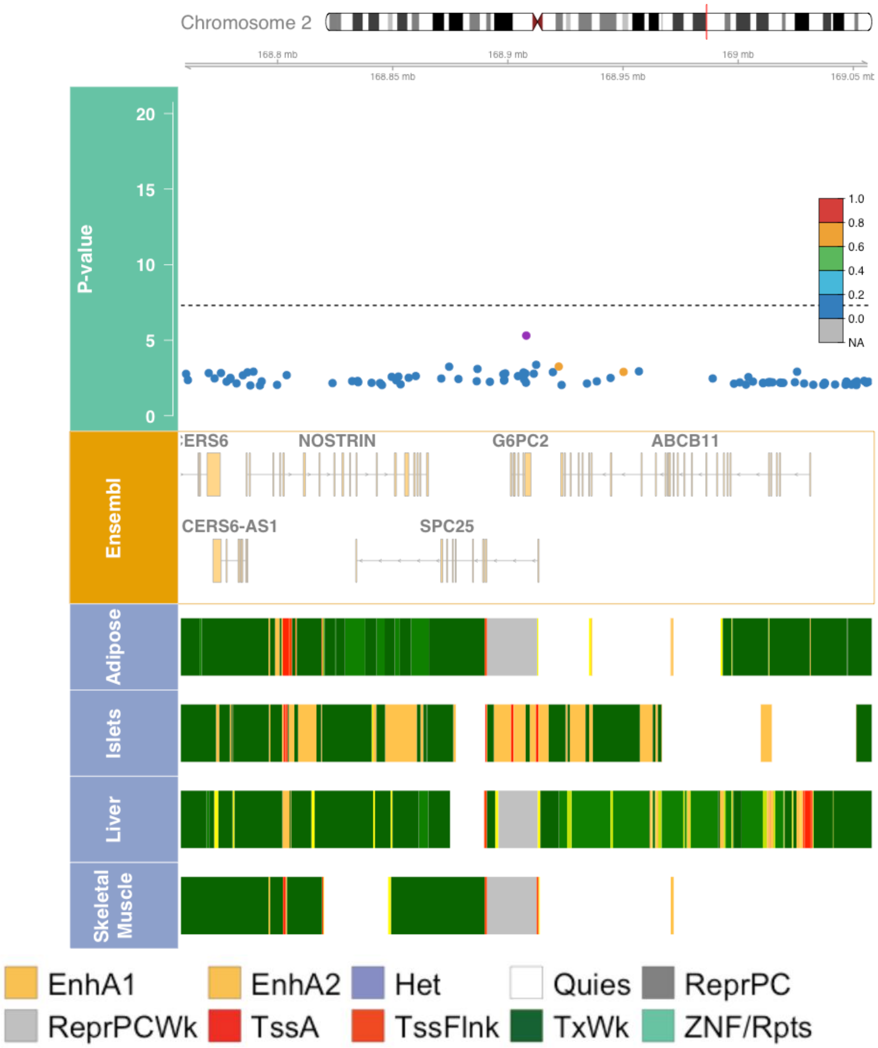

f. *GCK*, rs1799884, 7:44189469:C:T

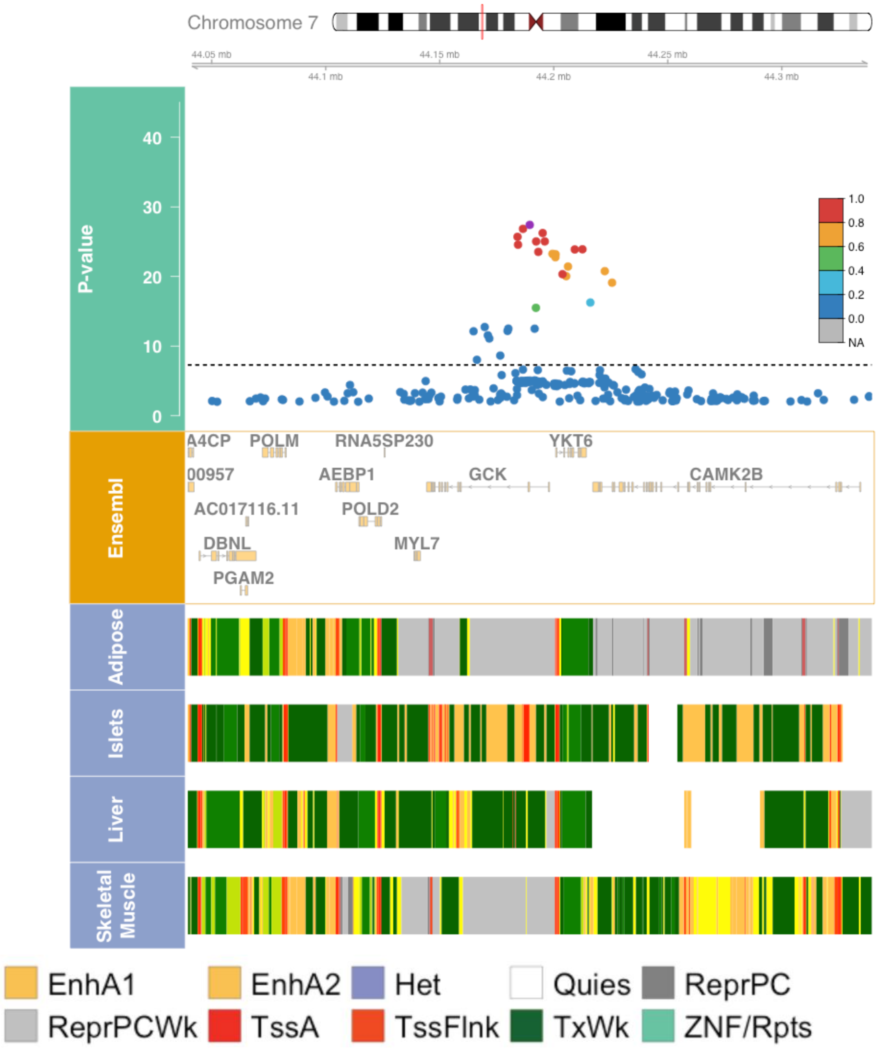

g. *GCKR*, rs1260326, 2:27508073:T:C

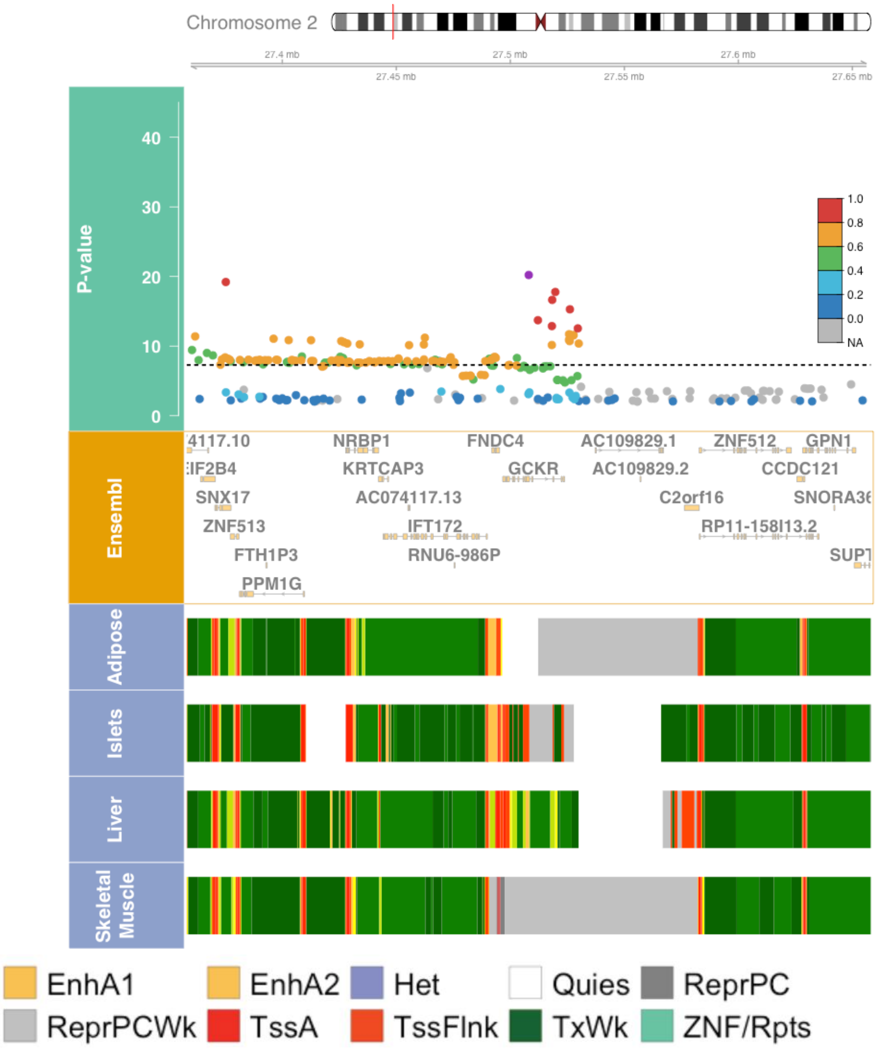

h. *FOXA2*, rs3833331, 20:22581688:A:AG

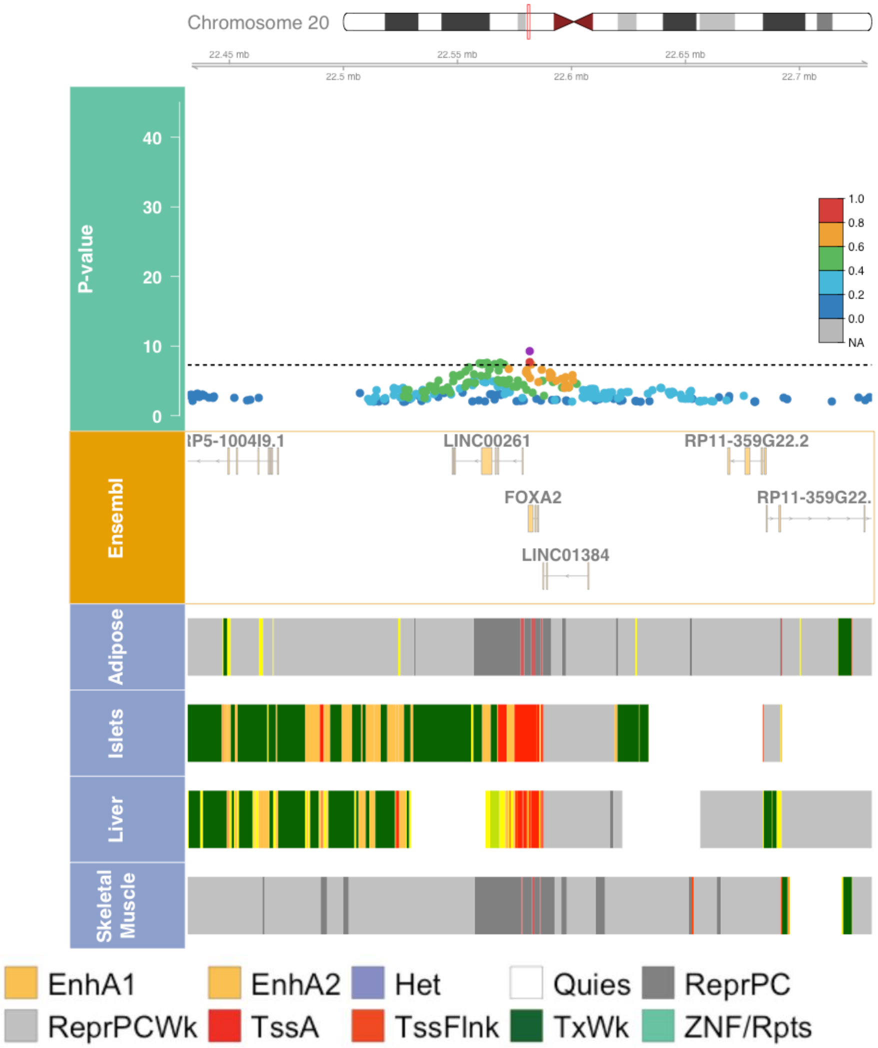

i. *SLC30A8*, rs35859536, 8:117179236:C:T

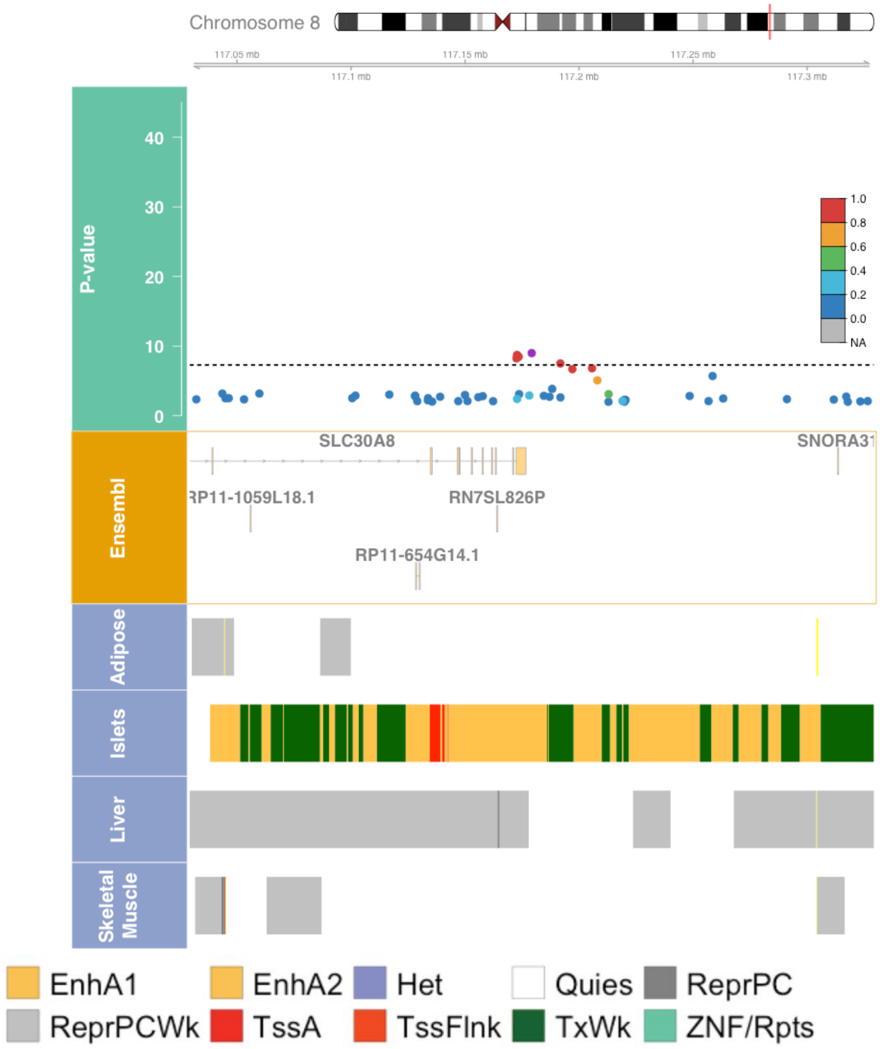

j. *SLC30A8*, rs542965166, 8:117258547:C:T

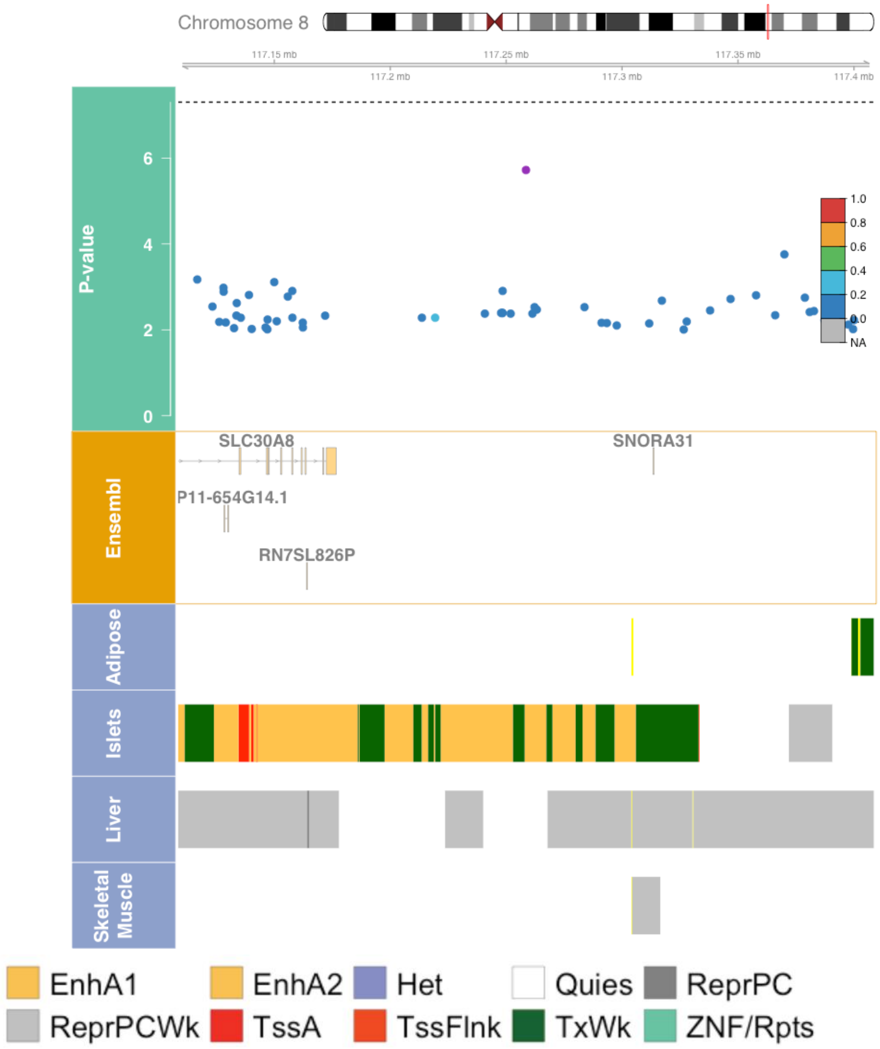

k. *APOB*, rs478588, 2:21074277:A:G

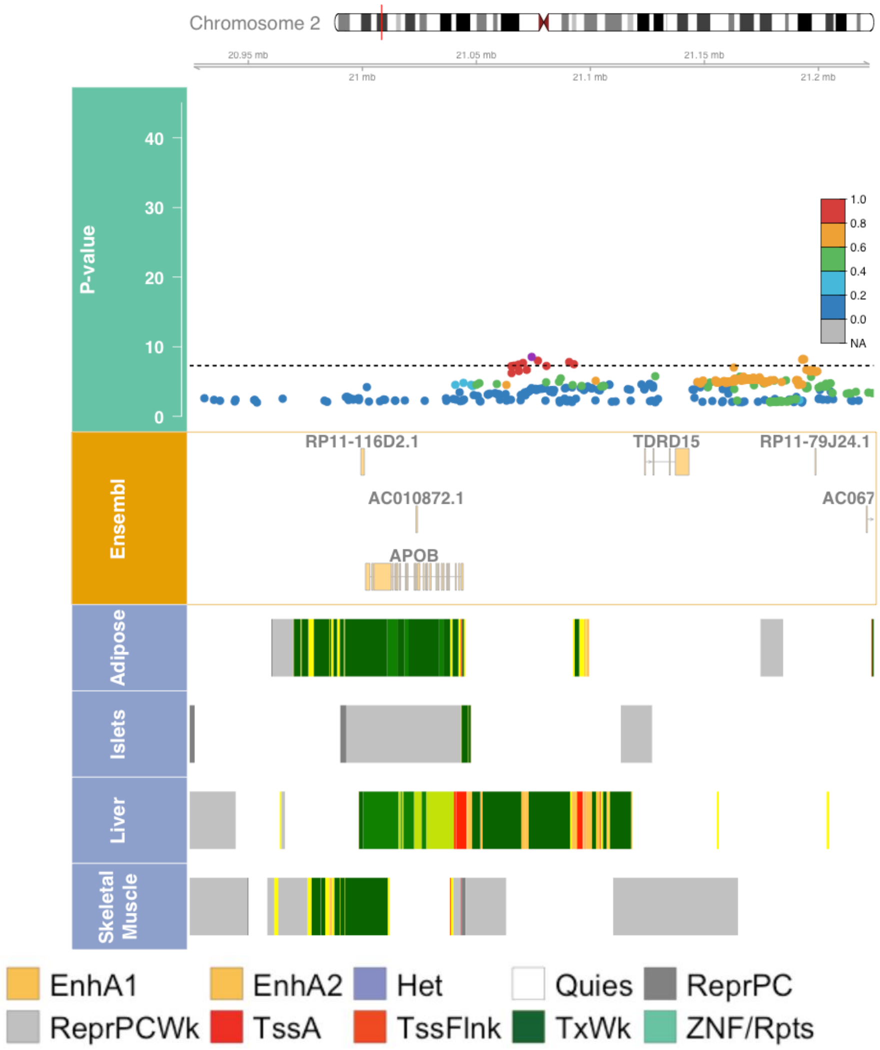

I. *TCF7L2*, rs7903146, 10:112998590:C:T

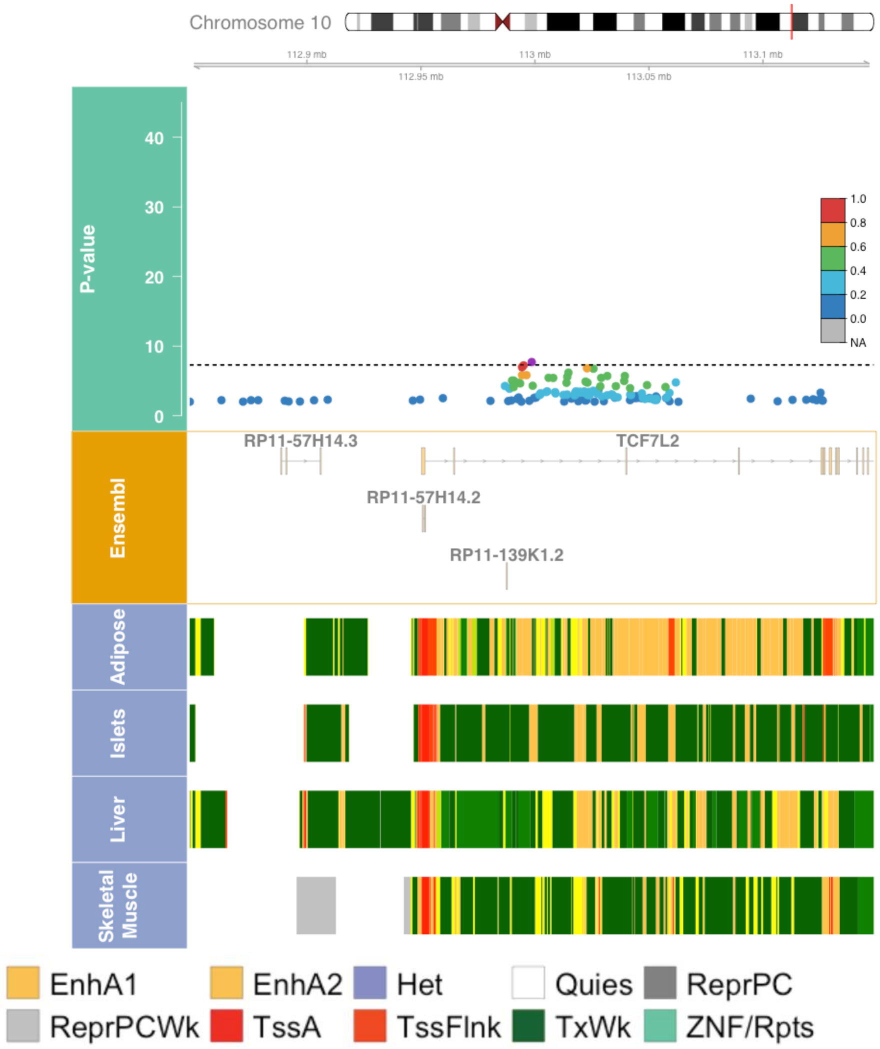

m. *ADCY5*, rs72964564, 3:123335923:A:C

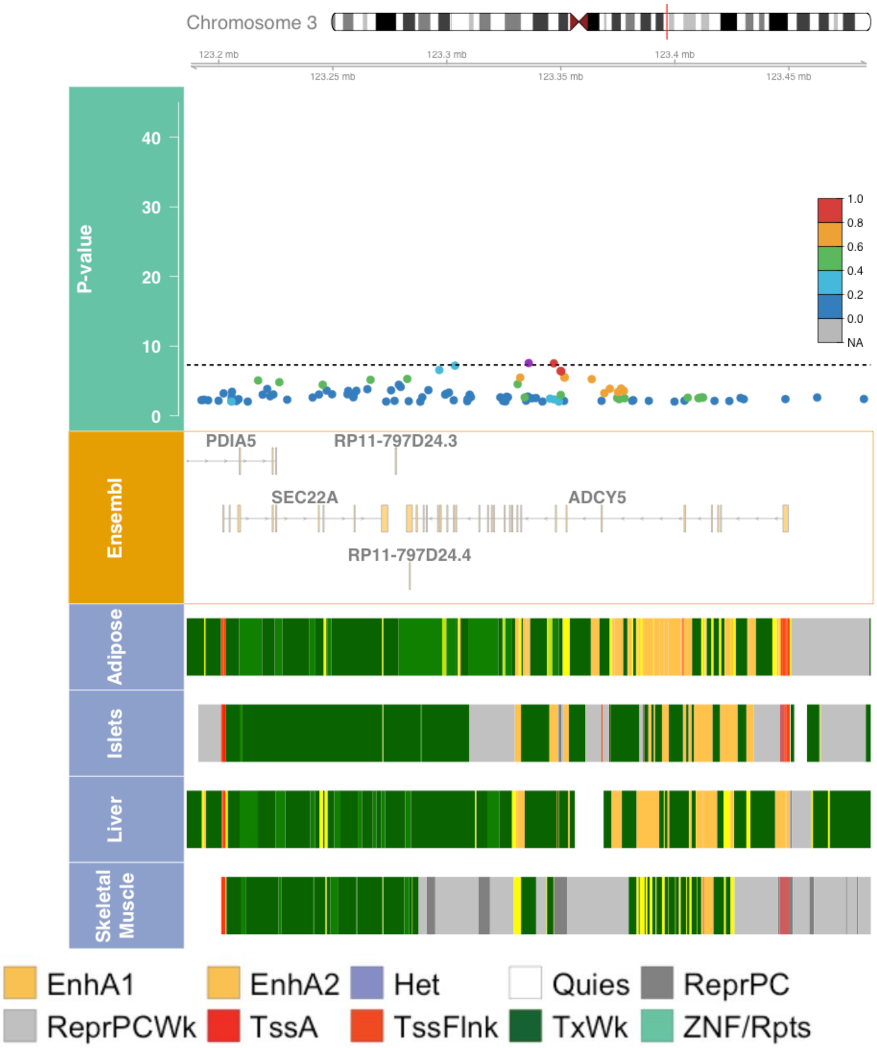

n. *GCKR*, rs1260326, 2:27508073:T:C

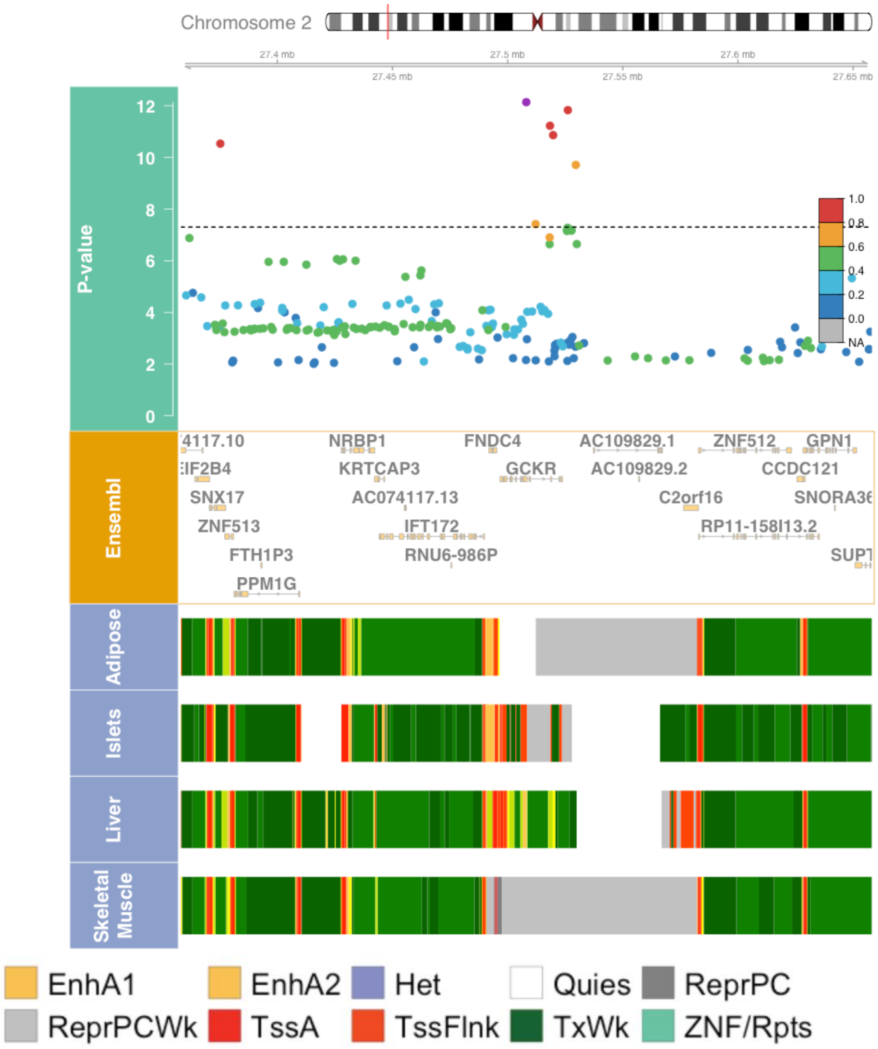

o. *PTPRT*, rs185250851, 20:42752773:G:A

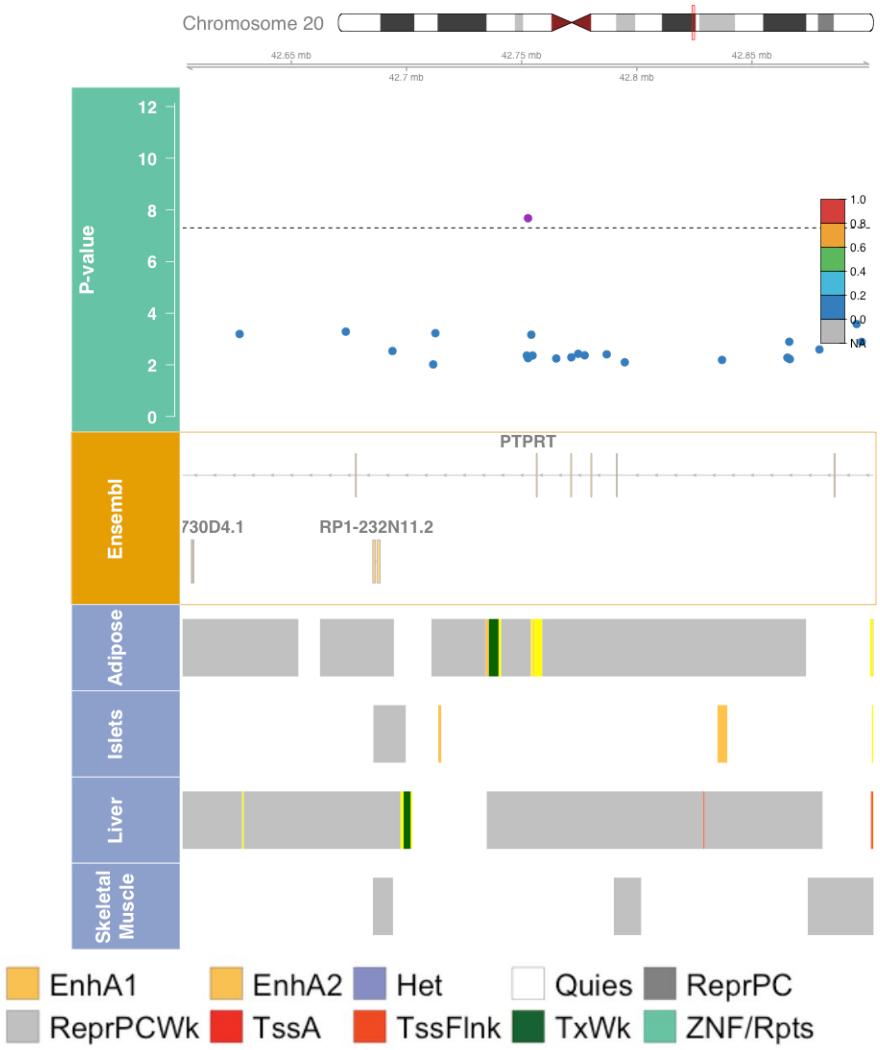

p. *PTPRT*, rs78618809, 20:43230137:C:T

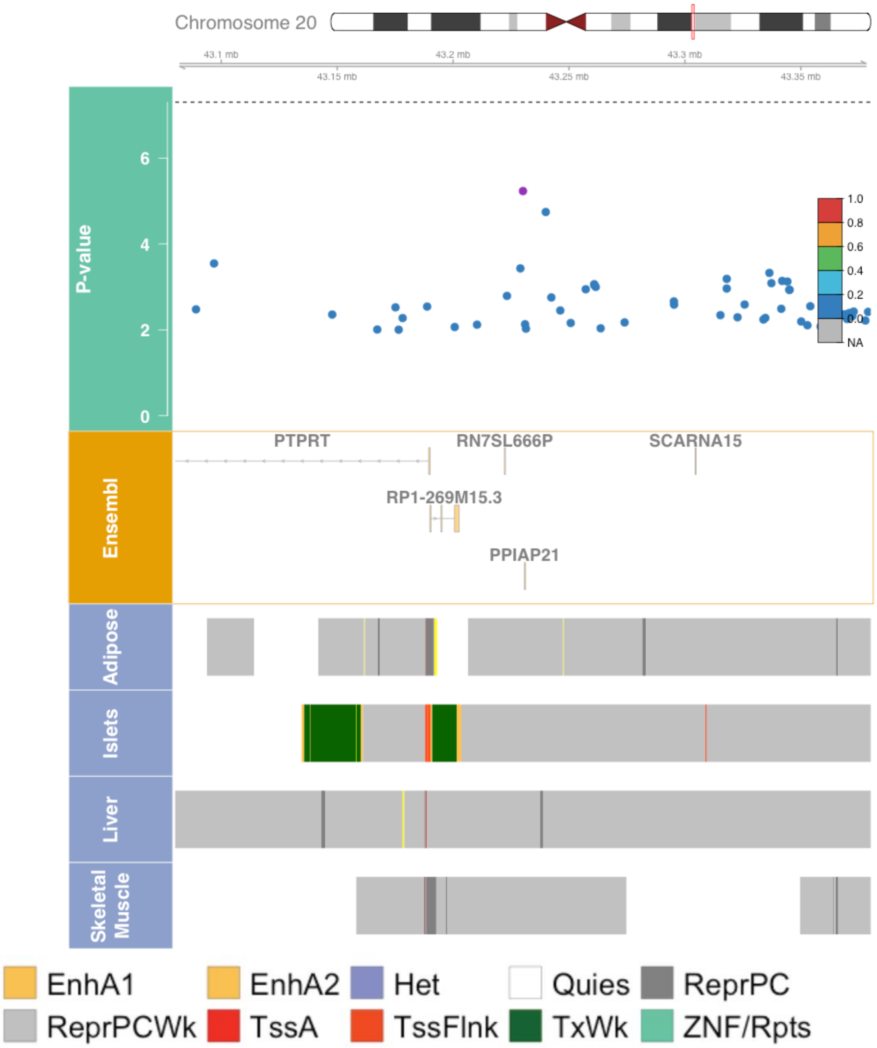

q. *ROBO1*, rs539973028, 3:79812347:C:A

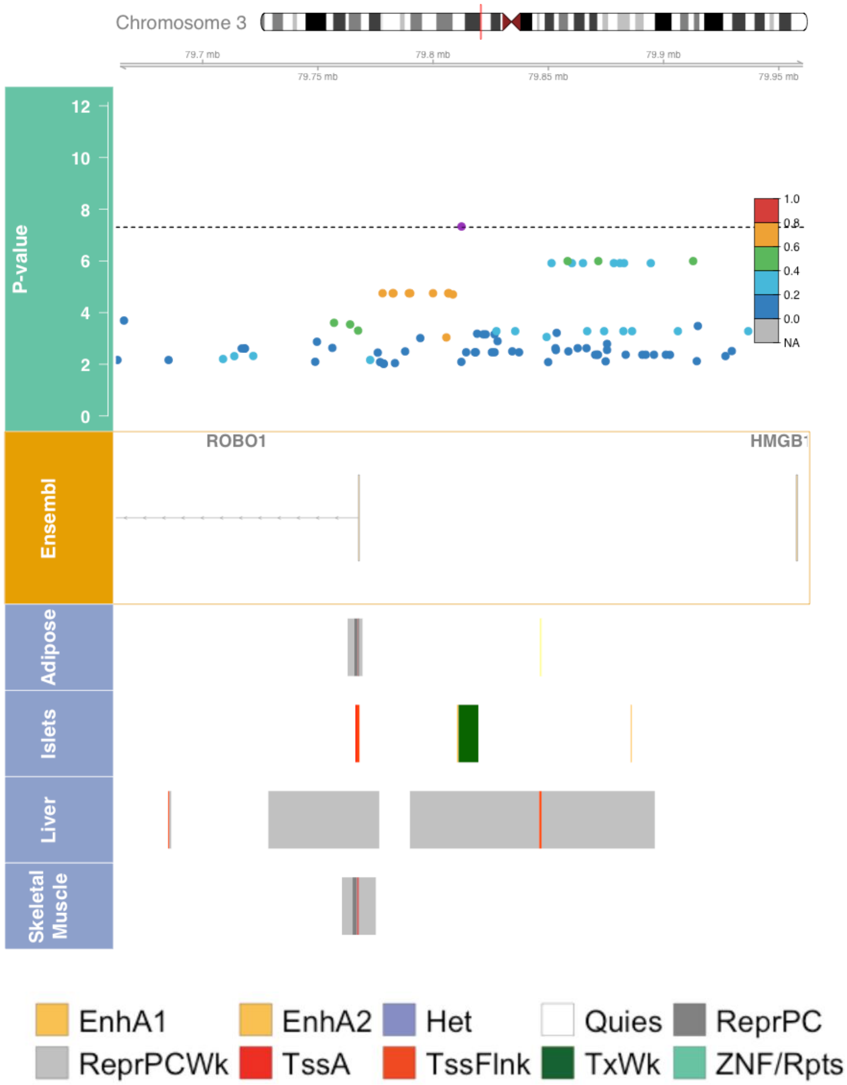

**Supplementary Figure 3.** Plot of associations with related traits of identified loci in single variant analysis. The plot shows a black indicator for traits that have been previously identified as associated; see also **Supplementary Table 8**.

| | FG | FI | HbA1c | T2D | BMI | WHR | HOMA-B |
|---|---|---|---|---|---|---|---|
| *MTNR1B* | ● | | ● | ● | ● | | ● |
| *G6PC2* | ● | | ● | | | | ● |
| *GCK* | ● | | ● | ● | | | ● |
| *GCKR* | ● | ● | | ● | ● | | |
| *FOXA2* | ● | | | ● | | | |
| *SLC30A8* | ● | | ● | ● | ● | | |
| *APOB* | | | | | | | |
| *TCF7L2* | ● | ● | ● | ● | ● | ● | |
| *ADCY5* | ● | | ● | ● | ● | ● | ● |
| *PTPRT* | | | | | ● | ● | |
| *ROBO1* | | | | | ● | ● | |

Abbreviations: FG, fasting glucose; FI, fasting insulin; HbA1c, hemoglobin A1c; T2D, Type 2 Diabetes; BMI, body mass index; WHR, waist-hip ratio; HOMA-B, homeostatic model assessment of beta-cell function.

**Supplementary Figure 4.** Manhattan plots for pooled region-based rare variant aggregate analysis with fasting glucose (a-d) and fasting insulin (e-h) in TOPMed. The Manhattan plot shows the -$\log_{10}$ *P-values* for the variant sets plotted at the starting chromosomal positions, using the burden test and the SKAT test.

a. Fasting Glucose, SKAT (1,1)

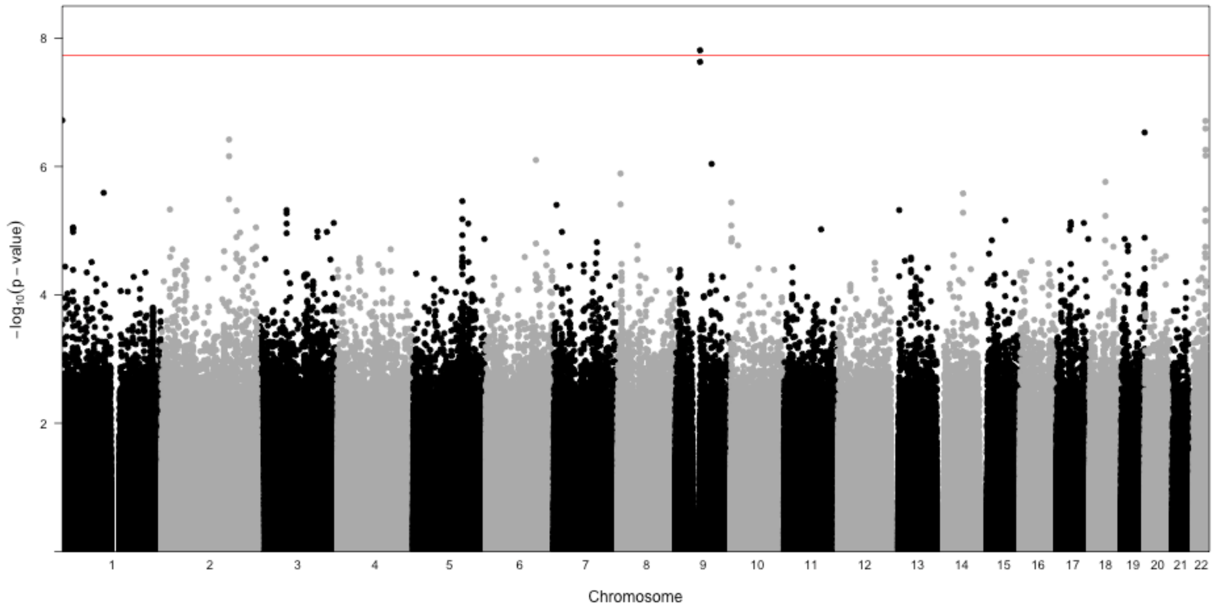


b. Fasting Glucose, SKAT (1,25)

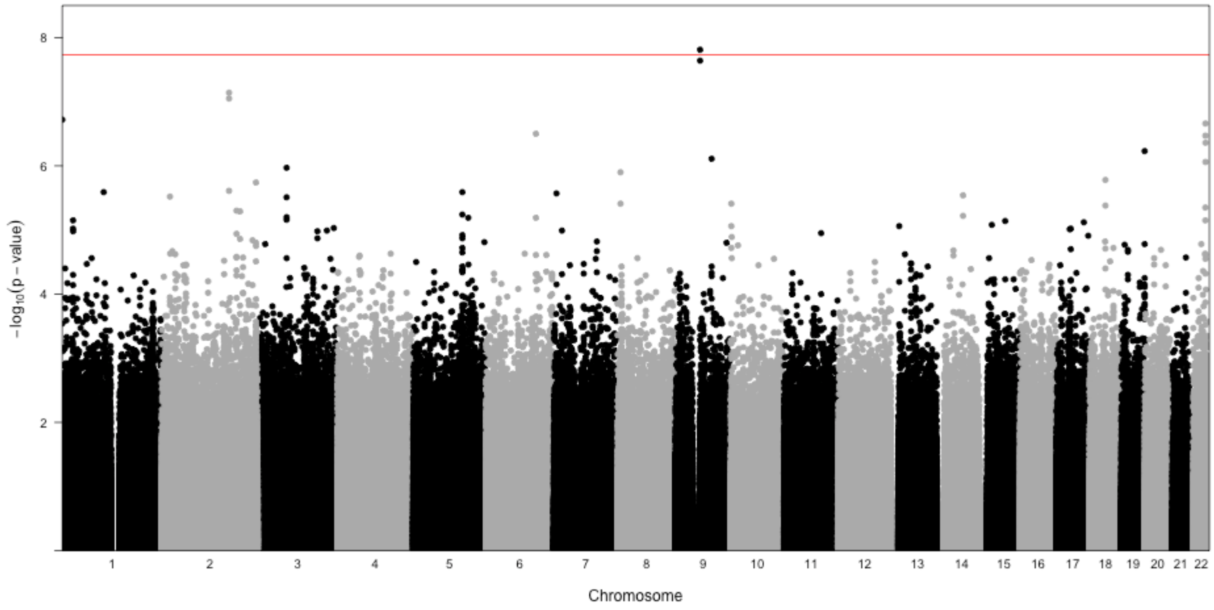

c. Fasting Glucose, Burden (1,1)

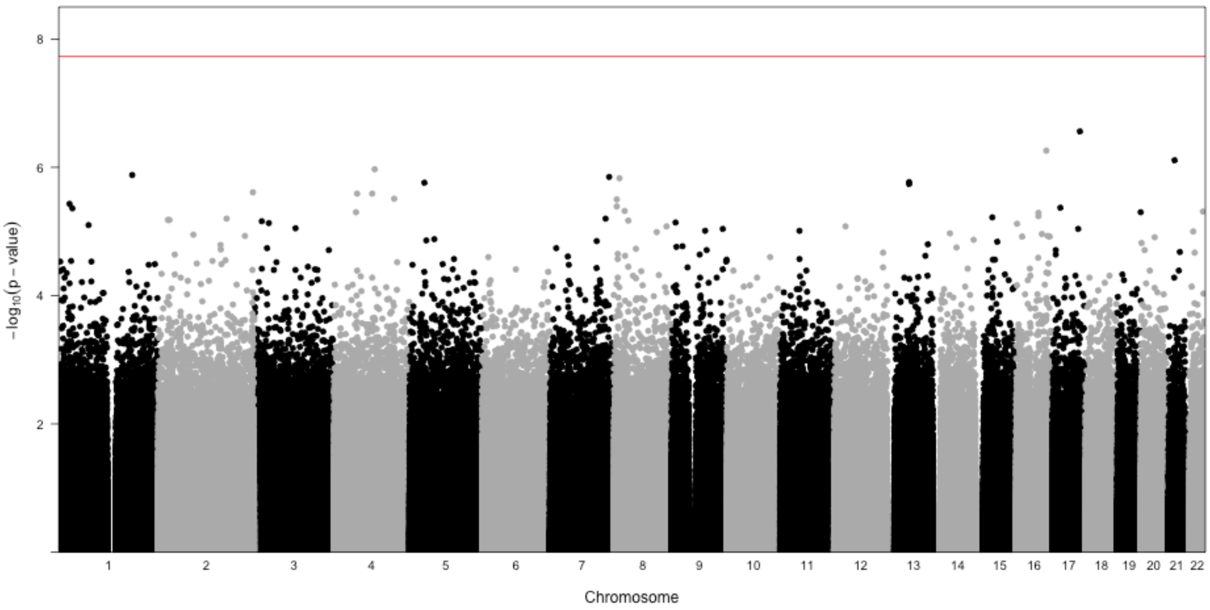


d. Fasting Glucose, Burden (1,25)

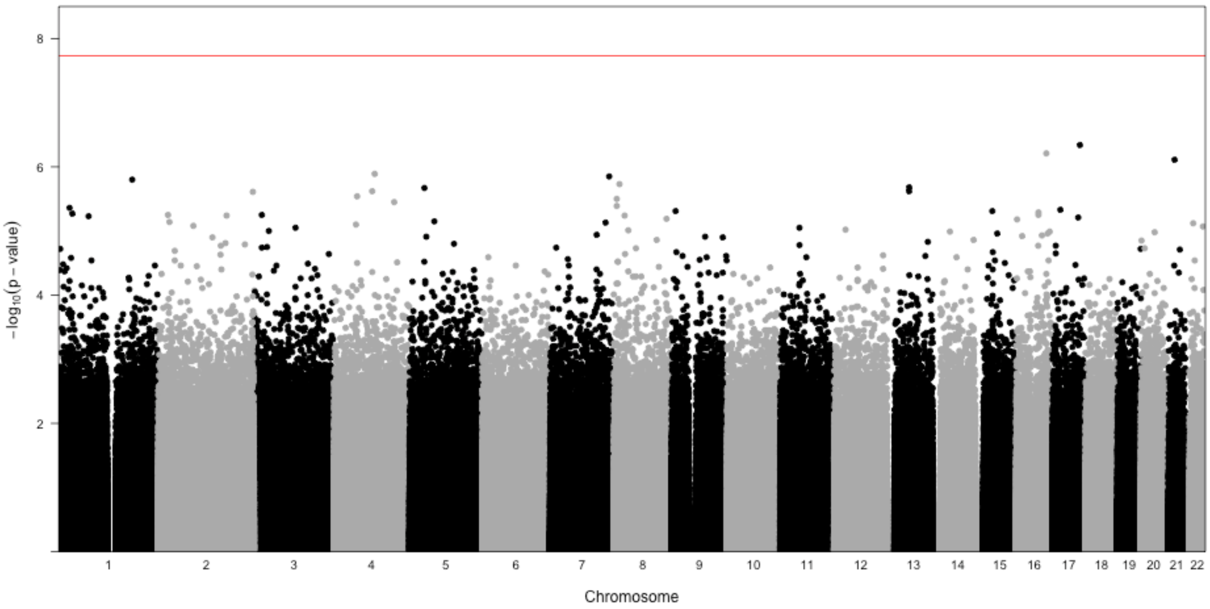

e. Fasting Insulin, SKAT (1,1)

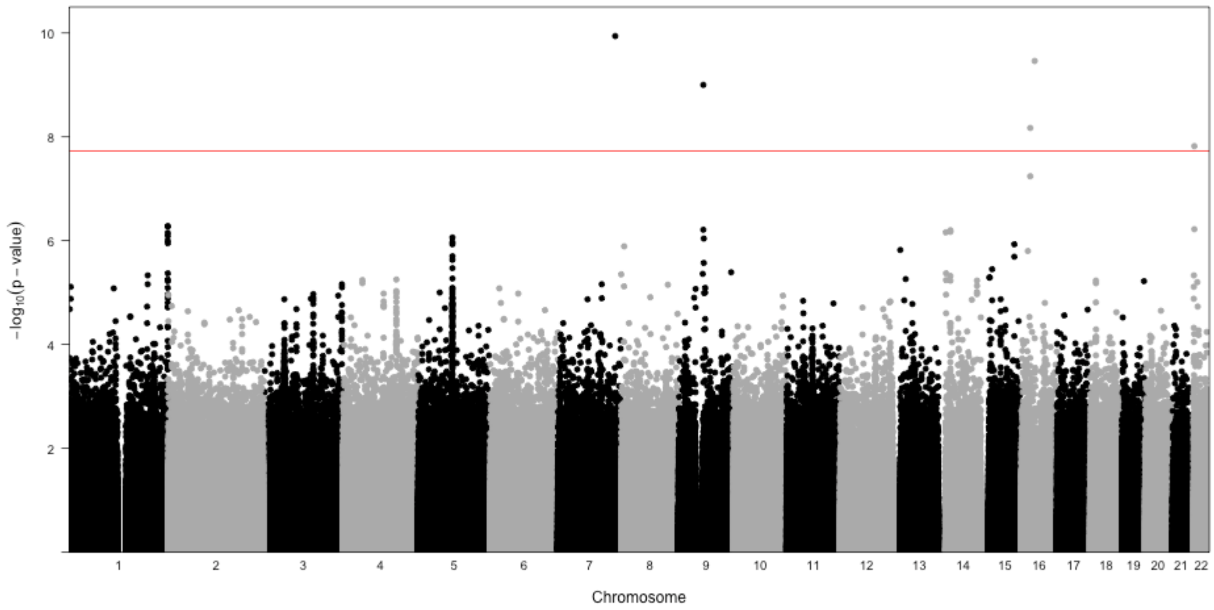


f. Fasting Insulin, SKAT (1,25)

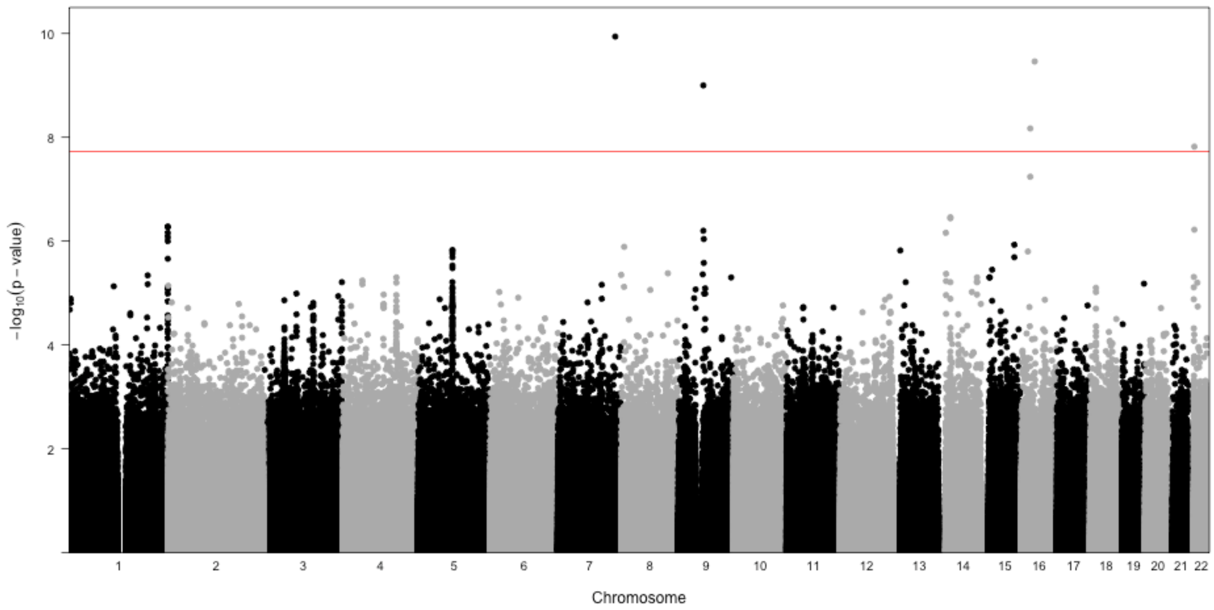

g. Fasting Insulin, Burden (1,1)

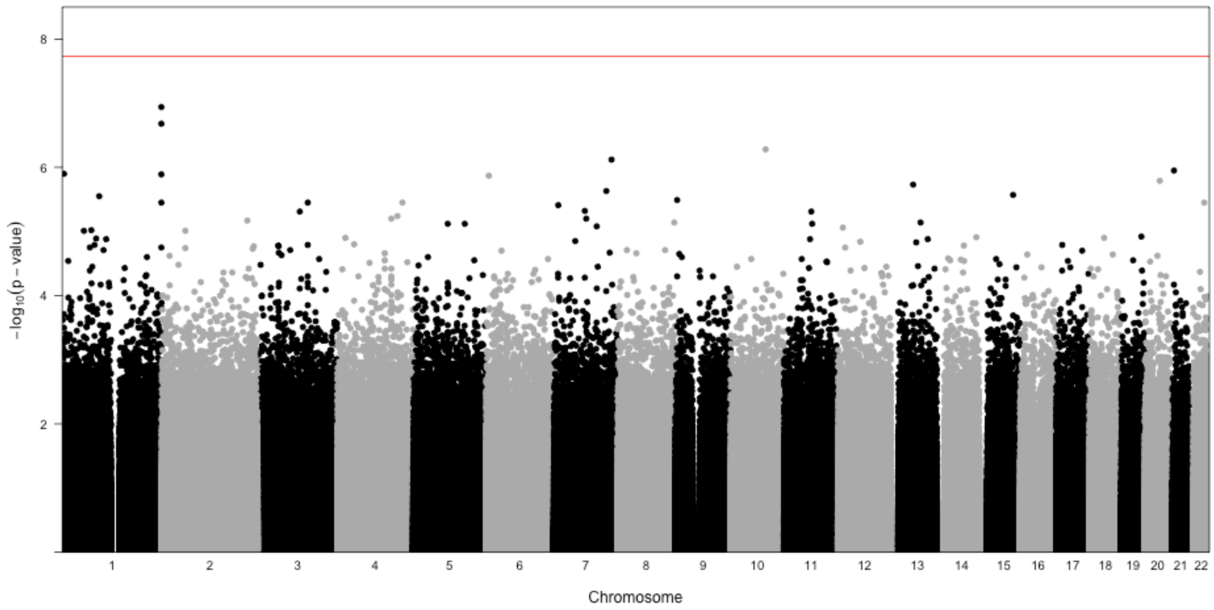


h. Fasting Insulin, Burden (1,25)

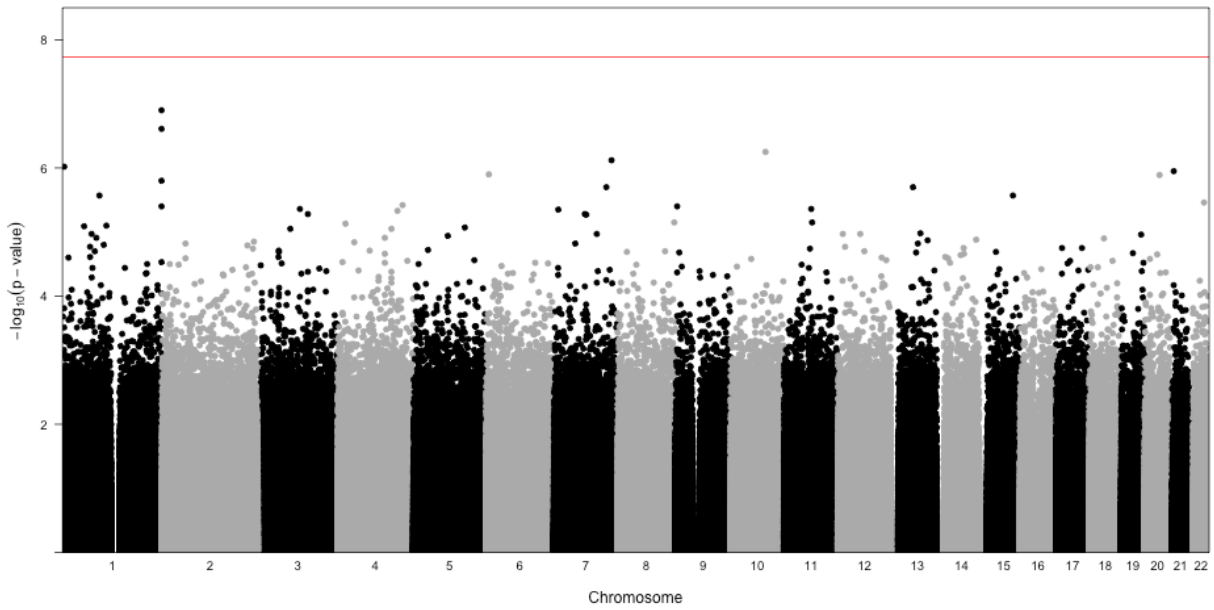

# Supplementary Tables

## Supplementary Table 1. Description of TOPMed studies included in glycemic trait analysis

| TOPMed Project | TOPMed Accession | Parent Study Accession | TOPMed Study | Study/Cohort Abbreviation | Country | Study Design | FG Unit | FG Source | FI Unit |
|---|---|---|---|---|---|---|---|---|---|
| Amish | phs000956 | | Genetics of Cardiometabolic Health in the Amish | Amish | USA | Community-based | mmol/l | Plasma | mU/l |
| AFGen - VTE | phs001211 | phs000280 | Trans-Omics for Precision Medicine Whole Genome Sequencing Project: ARIC | ARIC | USA | Population-based/ Case-ascertained | mmol/l | Serum | mU/l |
| CFS | phs000954 | phs000284 | The Cleveland Family Study (WGS) | CFS | USA | family-based | mmol/l | Plasma | mU/l |
| VTE | phs001368 | phs000287 | Cardiovascular Health Study | CHS | USA | Population-based | mmol/l | Plasma | pmol/L |
| AFGen, FHS | phs000974 | phs000007 | Whole Genome Sequencing and Related Phenotypes in the Framingham Heart Study | FHS | USA | Population-based | mmol/l | Plasma | mU/l |
| AA_CAC - GeneSTAR | phs001218 | phs001074 | GeneSTAR (Genetic Study of Atherosclerosis Risk) | GeneSTAR | USA | Family-based | mmol/l | Serum | pmol/L |
| AA_CAC - HyperGEN_GENOA | phs001345 | phs001238 | Genetic Epidemiology Network of Arteriopathy (GENOA) | GENOA | USA | Cohort of sibships enriched for hypertension | mmol/l | Plasma | pmol/L |
| GenSalt | phs001217 | phs000784 | Genetic Epidemiology Network of Salt Sensitivity (GenSalt) | GenSalt | China | Longitudinal family study | mmol/l | Plasma | N/A |
| GOLDN | phs001359 | phs000741 | Genetics of Lipid Lowering Drugs and Diet Network (GOLDN) | GOLDN | USA | population based | mg/dl | Plasma | mU/l |
| HyperGEN_GENOA | phs001293 | | HyperGEN - Genetics of Left Ventricular (LV) Hypertrophy | HyperGEN | USA | population based | mg/d | Plasma | mU/l |
| JHS | phs000964 | phs000286 | The Jackson Heart Study | JHS | USA | Population-based | mmol/l | Plasma | pmol/L |
| AA_CAC - MESA | phs001416 | phs000209 | MESA and MESA Family AA-CAC | MESA | USA | Population-based | mmol/l | Serum | mU/l |
| SAFS | phs001215 | | San Antonio Family Heart Study (WGS) | SAFS | USA | Pedigree-based random ascertainment | mmol/l | Plasma | pmol/l |
| Samoan | phs000972 | phs000914 | Genome-wide Association Study of Adiposity in Samoans | Samoan | Samoa | Population-based | mmol/l | Serum | mU/l |
| WHI | phs001237 | phs000200 | Women's Health Initiative (WHI) | WHI | USA | Population-based | mmol/l | Serum | pmol/L |

**Supplementary Table 2. Demographic characteristics of cohorts included in fasting glucose analysis**

| Study | N | Male (%) | Female (%) | Age (SD) | African (%) | Asian (%) | European (%) | Hispanic / LatinX (%) | Samoan (%) | FG (SD[a]) |
|---|---|---|---|---|---|---|---|---|---|---|
| Amish | 908 | 454 (50%) | 454 (50%) | 49.7 (16.5) | | | 908 (100%) | | | 4.78 (0.58) |
| ARIC | 2776 | 1308 (47.1%) | 1468 (52.9%) | 54.5 (5.7) | 148 (5.3%) | | 2628 (94.7%) | | | 5.43 (0.46) |
| CFS | 427 | 174 (40.7%) | 253 (59.3%) | 37.7 (18.1) | 236 (55.3%) | | 191 (44.7%) | | | 5.68 (0.52) |
| CHS | 60 | 23 (38.3%) | 37 (61.7%) | 72.9 (5.3) | 9 (15%) | | 51 (85%) | | | 5.43 (0.54) |
| FHS | 2896 | 1348 (46.5%) | 1548 (53.5%) | 48.5 (11.9) | | | 2896 (100%) | | | 5.23 (0.51) |
| GeneSTAR | 1371 | 563 (41.1%) | 808 (58.9%) | 42.7 (10.4) | 587 (42.8%) | | 784 (57.2%) | | | 4.99 (0.58) |
| GENOA | 807 | 247 (30.6%) | 560 (69.4%) | 56.3 (10.7) | 807 (100%) | | | | | 5.18 (0.6) |
| GenSalt | 1635 | 873 (53.4%) | 762 (46.6%) | 38.6 (9.5) | | 1635 (100%) | | | | 4.79 (0.58) |
| GOLDN | 807 | 382 (47.3%) | 425 (52.7%) | 46.6 (16.3) | | | 807 (100%) | | | 5.42 (0.5) |
| HyperGEN | 1349 | 516 (38.3%) | 833 (61.7%) | 44.8 (12.6) | 1349 (100%) | | | | | 5.17 (0.59) |
| JHS | 2321 | 889 (38.3%) | 1432 (61.7%) | 54 (13) | 2321 (100%) | | | | | 5.01 (0.5) |
| MESA | 3648 | 1770 (48.5%) | 1878 (51.5%) | 60.8 (9.9) | 815 (22.3%) | 478 (13.1%) | 1580 (43.3%) | 775 (21.2%) | | 4.95 (0.57) |
| SAFS | 1004 | 426 (42.4%) | 578 (57.6%) | 39.5 (15.7) | | | | 1004 (100%) | | 5.02 (0.55) |
| Samoan | 914 | 357 (39.1%) | 557 (60.9%) | 43.3 (11.1) | | | | | 914 (100%) | 4.93 (0.75) |
| WHI | 5884 | | 5884 (100%) | 66.7 (6.9) | 902 (15.3%) | 104 (1.8%) | 4668 (79.3%) | 210 (3.6%) | | 5.05 (0.5) |
| **Overall** | 26807 | 9330 (34.8%) | 17477 (65.2%) | 53.8 (14.3) | 7174 (26.8%) | 2217 (8.3%) | 14513 (54.1%) | 1989 (7.4%) | 914 (3.4%) | 5.09 (0.57) |

[a] SD, standard deviation

**Supplementary Table 3. Demographic characteristics of cohorts included in fasting insulin analysis**

| Study | N | Male (%) | Female (%) | Age (SD) | African (%) | Asian (%) | European (%) | Hispanic / LatinX (%) | Samoan (%) | FG (SD[a]) |
|---|---|---|---|---|---|---|---|---|---|---|
| Amish | 898 | 448 (49.9%) | 450 (50.1%) | 49.7 (16.6) | | | 898 (100%) | | | 2.18 (0.48) |
| ARIC | 2712 | 1278 (47.1%) | 1434 (52.9%) | 54.4 (5.7) | 135 (5%) | | 2577 (95%) | | | 2.11 (0.65) |
| CFS | 424 | 173 (40.8%) | 251 (59.2%) | 37.7 (18.1) | 236 (55.7%) | | 188 (44.3%) | | | 2.28 (0.6) |
| CHS | 59 | 23 (39%) | 36 (61%) | 73 (5.3) | 9 (15.3%) | | 50 (84.7%) | | | 2.6 (0.44) |
| FHS | 2730 | 1275 (46.7%) | 1455 (53.3%) | 48.6 (12) | | | 2730 (100%) | | | 3.31 (0.39) |
| GeneSTAR | 449 | 175 (39%) | 274 (61%) | 48.2 (7.9) | 257 (57.2%) | | 192 (42.8%) | | | 2.05 (0.65) |
| GENOA | 806 | 246 (30.5%) | 560 (69.5%) | 56.3 (10.7) | 806 (100%) | | | | | 2.24 (0.75) |
| GOLDN | 805 | 380 (47.2%) | 425 (52.8%) | 46.6 (16.3) | | | 805 (100%) | | | 2.46 (0.47) |
| HyperGEN | 1347 | 517 (38.4%) | 830 (61.6%) | 44.8 (12.6) | 1347 (100%) | | | | | 2.01 (0.7) |
| JHS | 2310 | 888 (38.4%) | 1422 (61.6%) | 54 (13) | 2310 (100%) | | | | | 2.62 (0.53) |
| MESA | 3648 | 1770 (48.5%) | 1878 (51.5%) | 60.8 (9.9) | 815 (22.3%) | 478 (13.1%) | 1580 (43.3%) | 775 (21.2%) | | 1.65 (0.62) |
| SAFS | 660 | 257 (38.9%) | 403 (61.1%) | 43.9 (15.2) | | | | 660 (100%) | | 2.76 (0.59) |
| Samoan | 914 | 357 (39.1%) | 557 (60.9%) | 43.3 (11.1) | | | | | 914 (100%) | 2.41 (0.79) |
| WHI | 5449 | | 5449 (100%) | 66.5 (7) | 888 (16.3%) | 94 (1.7%) | 4261 (78.2%) | 206 (3.8%) | | 2.01 (0.59) |
| Overall | 23211 | 7787 (33.5%) | 15424 (66.5%) | 55.5 (13.5) | 6803 (29.3%) | 572 (2.5%) | 13281 (57.2%) | 1641 (7.1%) | 914 (3.9%) | 2.25 (0.76) |

[a] SD, standard deviation

## Supplementary Table 4. Haplotype analysis of the *G6PC2* locus

| Marginal Effects with Respect to Effect Allele | | | | | | | | | |
|---|---|---|---|---|---|---|---|---|---|
| Position[a] | Signal | RSID | EA[b] | EAF[c] | EAC[d] | N | P-Value | Beta | SE |
| 2:168906638:T:C | GWAS, Primary signal | rs560887 | C | 0.82 | 43730 | 26807 | 6.8E-37 | 0.0717 | 0.0057 |
| 2:168900420:A:G | Secondary signal | rs540524 | A | 0.66 | 35607 | 26807 | 8.4E-02 | 0.0080 | 0.0046 |
| 2:168907981:T:C | Tertiary/Rare Variant signal | rs2232326 | T | 0.99 | 53345 | 26807 | 3.8E-05 | 0.1346 | 0.0327 |
| 2:168907631:A:C | Rare Variant signal, Mahajan - p.Tyr207Ser | rs2232323 | A | 0.99 | 53302 | 26807 | 1.8E-04 | 0.1038 | 0.0277 |
| 2:168906752:C:T | Mahajan - p.His177Tyr | rs138726309 | C | 0.99 | 53511 | 26807 | 2.9E-02 | 0.1000 | 0.0458 |
| 2:168907666:G:C | Mahajan - p.Val219Leu | rs492594 | C | 0.41 | 22387 | 26807 | 9.9E-01 | 0.0001 | 0.0044 |

| Previous Haplotype Variants | | | | Secondary | Tertiary/Rare Variant | | | |
|---|---|---|---|---|---|---|---|---|
| 2:168906638 rs560887 | 2:168906752 rs138726309 | 2:168907631 rs2232323 | 2:168907666 rs492594 | 2:168900420 rs540524 | 2:168907981 rs2232326 | Frequency | Beta | SE |
| C | C | A | C[e] | G[e] | T | 0.32 | Baseline | |
| | | | | A | T | 0.01 | 0.03 | 3.9E-03 |
| | | | | G[e] | C[e] | 0.001 | -0.15 | 2.4E-05 |
| T[e] | C | A | G | A | T | 0.19 | -0.05 | 5.8E-03 |
| C | C | A | G | A | C[e] | 0.003 | -0.09 | 4.1E-05 |
| | | | | A | T | 0.37 | 0.04 | 4.9E-03 |
| | | | | G[e] | T | 0.02 | 0.04 | 1.6E-04 |
| T[e] | C | C[e] | G | A | T | 0.006 | -0.11 | 7.5E-05 |
| C | T[e] | A | C[e] | A | T | 0.002 | -0.09 | 3.3E-05 |

[a] Format: Chromosome, Position (Hg38), Reference Allele, Alternate Allele
[b] Effect Allele
[c] Effect Allele Frequency
[d] Effect Allele Count
[e] Glucose Lowering

## Supplementary Table 5. Validation lookups of significant and suggestive novel signals in the UKBB

| Trait | Gene | Chr[a] | Position (HG38) | Ref[b] | Alt[c] | rsID | Imputation $r^2$ | N | ALT.AC[d] | MAC[e] | MAF[f] | P-value | Beta (Alt allele) | SE[g] Beta |
|---|---|---|---|---|---|---|---|---|---|---|---|---|---|---|
| Fasting Glucose | *MTNR1B* | 11 | 9288161 | G | A | rs73560545 | 1.00 | 12,798 | 3459 | 3459 | 0.14 | 9.3E-01 | -7.7E-04 | 9.4E-03 |
| | *SLC30A8* | 8 | 117179236 | C | T | rs35859536 | 1.00 | 12,798 | 8105 | 8105 | 0.32 | 4.6E-05 | -2.8E-02 | 6.9E-03 |
| | *APOB* | 2 | 21074277 | A | G | rs478588 | 1.00 | 12,798 | 20539 | 5057 | 0.80 | 1.1E-02 | 2.0E-02 | 8.1E-03 |
| | *ADCY5* | 3 | 123335923 | A | C | rs72964564 | 0.99 | 12,798 | 6438 | 6438 | 0.25 | 1.0E-07 | -3.9E-02 | 7.4E-03 |
| | *HS6ST3/UGGT2* | 13 | 96407609 | A | G | rs1328056 | 1.00 | 12,798 | 1536 | 1536 | 0.06 | 5.7E-01 | 7.8E-03 | 1.4E-02 |
| | *CTD-2199O4.4/ ATPSCKMT/CCT5* | 5 | 10169711 | T | C | rs13361160 | 1.00 | 12,798 | 11095 | 11095 | 0.43 | 6.3E-01 | 3.1E-03 | 6.4E-03 |

[a] Chromosome, [b] Reference Allele, [c] Alternative Allele, [d] Alternative Allele Count, [e] Minor Allele Count, [f] Minor Allele Frequency, [g] Standard Error

## Supplementary Table 6. Validation lookups of significant and suggestive novel signals in METSIM cohorts

| Trait | Gene | Chr[a] | Position (HG38) | Ref[b] | Alt[c] | rsID | Imputation $r^2$ | N | ALT.AC[d] | MAC[e] | MAF[f] | P-value | Beta (Alt allele) | SE[g] Beta |
|---|---|---|---|---|---|---|---|---|---|---|---|---|---|---|
| Fasting Glucose | *MTNR1B* | 11 | 92884161 | G | A | rs73560545 | 0.99 | 10058 | 3201.42 | 3201 | 0.16 | 0.853 | 0.004 | 0.021 |
| | *SLC30A8* | 8 | 117179236 | C | T | rs35859536 | 0.99 | 10058 | 7898.28 | 7898 | 0.39 | 0.000 | -0.073 | 0.016 |
| | *SLC30A8* | 8 | 117258547 | C | T | rs542965166 | 0.01 | 10058 | 0.29 | 0.29 | 0.00001 | NA | NA | NA |
| | *APOB* | 2 | 21074277 | A | G | rs478588 | 0.99 | 10058 | 15779.39 | 4336. | 0.22 | 0.128 | -0.029 | 0.019 |
| | *ADCY5* | 3 | 123335923 | A | C | rs72964564 | 0.99 | 10058 | 3231.05 | 3231 | 0.16 | 0.071 | -0.037 | 0.021 |
| | *HS6ST3* | 13 | 96407609 | A | G | rs1328056 | 0.95 | 10058 | 1425.58 | 1425 | 0.07 | 0.526 | 0.019 | 0.030 |
| | *CTD-2199C04.4* | 5 | 10169711 | T | C | rs13361160 | 0.99 | 10058 | 8288.41 | 8288 | 0.41 | 0.875 | 0.002 | 0.015 |
| Fasting Insulin | *PTPRT* | 20 | 42752773 | G | A | rs185250851 | 0.98 | 10058 | 8.77 | 8 | 0.0004 | 0.051 | 0.351 | 0.180 |
| | *PTPRT* | 20 | 43230137 | C | T | rs78618809 | 0.29 | 10058 | 0.56 | 0.56 | 0.00003 | 0.514 | -0.862 | 1.321 |
| | *ROBO1* | 3 | 79812347 | C | A | rs539973028 | 0 | 10058 | 0 | 0 | 0 | NA | NA | NA |
| | *LINC00704, LINC00705* | 10 | 4656482 | GAAAAT | G | rs775018107 | 0.05 | 10058 | 0.82 | 0.82 | 0.00004 | 0.575 | -0.870 | 1.553 |
| | *RP11/IGSF11* | 3 | 118656074 | T | G | rs117592405 | 0.99 | 10058 | 131.47 | 131 | 0.007 | 0.438 | -0.036 | 0.047 |

[a] Chromosome, [b] Reference Allele, [c] Alternative Allele, [d] Alternative Allele Count, [e] Minor Allele Count, [f] Minor Allele Frequency, [g] Standard Error

## Supplementary Table 7. Validation lookups of novel suggestive signals in the Samoan cohort

| Trait | Gene | Chr[a] | Position (HG38) | Ref[b] | Alt[c] | rsID | Imputation $r^2$ | N | ALT.AC[d] | MAC[e] | MAF[f] | P-value | Beta (Alt allele) | SE[g] Beta |
|---|---|---|---|---|---|---|---|---|---|---|---|---|---|---|
| Fasting Insulin | *RP11/IGSF11* | 3 | 118656074 | T | G | rs117592405 | 0.98 | 1401 | 22 | 22 | 0.008 | 0.089 | -0.233 | 0.137 |

[a] Chromosome, [b] Reference Allele, [c] Alternative Allele, [d] Alternative Allele Count, [e] Minor Allele Count, [f] Minor Allele Frequency, [g] Standard Error

## Supplementary Table 8. Enrichment of Chromatin States at significant and suggestive Fasting Glucose signals

| Tissue | Chromatin State | 1000G Reference Population for LD | #SNPs in Chrom State | Expected #SNPS in Chrom State | P-Value |
|---|---|---|---|---|---|
| Islets | Active Enhancer 2 | AF | 6 | 1.4 | 0.0011 |
| Islets | Active Enhancer 2 | AS | 7 | 2.6 | 0.0036 |
| Islets | Active Enhancer 2 | EU | 8 | 2.8 | 0.0014 |
| Islets | Active Enhancer 2 | HS | 7 | 2.6 | 0.0056 |
| Islets | Weak Transcription | AF | 10 | 5.2 | 0.0064 |
| Islets | Weak Transcription | AS | 12 | 6.5 | 0.0013 |
| Islets | Weak Transcription | EU | 11 | 6.7 | 0.013 |
| Islets | Weak Transcription | HS | 12 | 6.3 | 0.0012 |
| Islets | Genic Enhancer | HS | 3 | 0.6 | 0.014 |
| Islets | Genic Enhancer | AS | 1 | 0.0 | 0.022 |

## Supplementary Table 9. Number of rare variant sets by phenotype and set-type

| Mask | Number of tests - FG | Number of tests - FI |
|---|---|---|
| Enhancer | 58677 | 58642 |
| Missense | 18256 | 18237 |
| Putative loss of function | 13723 | 13161 |
| Promoter | 20525 | 20496 |
| Synonymous | 18199 | 18182 |
| All Masks | 129380 | 128718 |

**Supplementary Data 1. 95% Credible Sets for all Reported Loci**
**See Supplementary Data Excel Sheet**

**Supplementary Data 2. 99% Credible Sets for Reported Loci from Pooled Analysis with <50 Variants**
**See Supplementary Data Excel Sheet**

**Supplementary Data 3. References for previous identification of variants in this study**
**See Supplementary Data Excel Sheet**

**Supplementary Data 4. Reference for previous identification of gene regions in this study**
**See Supplementary Data Excel Sheet**

**Supplementary Data 5. Lookups in related traits of identified loci in TOPMed projects**
**See Supplementary Data Excel Sheet**

**Supplementary Data 6. Significant Results of genome wide Fasting Glucose gene-centric rare variant aggregate tests**
**See Supplementary Data Excel Sheet**

**Supplementary Data 7. Significant Results of genome wide Fasting Glucose genetic-regions (sliding window) rare variant aggregate tests**
**See Supplementary Data Excel Sheet**

**Supplementary Data 8. Significant Results of genome wide log Fasting Insulin gene-centric rare variant aggregate tests.**
**See Supplementary Data Excel Sheet**

**Supplementary Data 9. Significant Results of genome wide log Fasting Insulin genetic-regions (sliding window) rare variant aggregate tests**
**See Supplementary Data Excel Sheet**
