# Supplementary material for: Whole genome sequence association analysis of fasting glucose and fasting insulin levels in diverse cohorts from the NHLBI TOPMed program
Source: Commun Biol. 2022 Jul 28;5:756. doi: 10.1038/s42003-022-03702-4 (PMC9334637; doi:10.1038/s42003-022-03702-4)
Supplement: Supplementary file 2 — Description of Additional Supplementary Files [file 42003_2022_3702_MOESM2_ESM.pdf]

Description of Additional Supplementary Files

File name: Supplementary Data 1
Description: 95% Credible Sets for all reported Loci.

File name: Supplementary Data 2
Description: 99% Credible Sets for Reported Loci from Pooled Analysis with <50 Variants.

File name: Supplementary Data 3
Description: References for previous identification of variants in this study.

File name: Supplementary Data 4
Description: Reference for previous identification of gene regions in this study.

File name: Supplementary Data 5
Description: Lookups in related traits of identified loci in TOPMed projects.

File name: Supplementary Data 6
Description: Significant Results of genome wide Fasting Glucose gene-centric rare variant aggregate tests.

File name: Supplementary Data 7
Description: Significant Results of genome wide Fasting Glucose genetic-regions (sliding window) rare variant aggregate tests.

File name: Supplementary Data 8
Description: Significant Results of genome wide log Fasting Insulin gene-centric rare variant aggregate tests.

File name: Supplementary Data 9
Description: Significant Results of genome wide log Fasting Insulin genetic-regions (sliding window) rare variant aggregate tests.
