# Supplementary material for: Whole genome sequence association analysis of fasting glucose and fasting insulin levels in diverse cohorts from the NHLBI TOPMed program
Source: Commun Biol. 2022 Jul 28;5:756. doi: 10.1038/s42003-022-03702-4 (PMC9334637; doi:10.1038/s42003-022-03702-4)
Supplement: Supplementary file 4 — Reporting Summary [file 42003_2022_3702_MOESM4_ESM.pdf]

nature research

Corresponding author(s): Alisa K Manning and Jennifer Wessel

Last updated by author(s): July 10, 2022

Reporting Summary

Nature Research wishes to improve the reproducibility of the work that we publish. This form provides structure for consistency and transparency in reporting. For further information on Nature Research policies, see our Editorial Policies and the Editorial Policy Checklist.

Statistics

For all statistical analyses, confirm that the following items are present in the figure legend, table legend, main text, or Methods section.

n/a	Confirmed	
☐	☒	The exact sample size (*n*) for each experimental group/condition, given as a discrete number and unit of measurement
☐	☒	A statement on whether measurements were taken from distinct samples or whether the same sample was measured repeatedly
☐	☒	The statistical test(s) used AND whether they are one- or two-sided<br>*Only common tests should be described solely by name; describe more complex techniques in the Methods section.*
☐	☒	A description of all covariates tested
☐	☒	A description of any assumptions or corrections, such as tests of normality and adjustment for multiple comparisons
☐	☒	A full description of the statistical parameters including central tendency (e.g. means) or other basic estimates (e.g. regression coefficient) AND variation (e.g. standard deviation) or associated estimates of uncertainty (e.g. confidence intervals)
☐	☒	For null hypothesis testing, the test statistic (e.g. *F*, *t*, *r*) with confidence intervals, effect sizes, degrees of freedom and *P* value noted<br>*Give P values as exact values whenever suitable.*
☒	☐	For Bayesian analysis, information on the choice of priors and Markov chain Monte Carlo settings
☒	☐	For hierarchical and complex designs, identification of the appropriate level for tests and full reporting of outcomes
☐	☒	Estimates of effect sizes (e.g. Cohen's *d*, Pearson's *r*), indicating how they were calculated

Our web collection on statistics for biologists contains articles on many of the points above.

Software and code

Policy information about availability of computer code

Data collection	The workflows utilized in this analysis are available in a public github repository (version 1.4.1; https://dockstore.org/workflows/github.com/AnalysisCommons/genesis_wdl/genesis_GWAS:v1_4_1?tab=info) from the Analysis Commons consortium.
Data analysis	The workflows utilized in this analysis are available in a public github repository (version 1.4.1; https://dockstore.org/workflows/github.com/AnalysisCommons/genesis_wdl/genesis_GWAS:v1_4_1?tab=info) from the Analysis Commons consortium.

For manuscripts utilizing custom algorithms or software that are central to the research but not yet described in published literature, software must be made available to editors and reviewers. We strongly encourage code deposition in a community repository (e.g. GitHub). See the Nature Research guidelines for submitting code & software for further information.

Data

Policy information about availability of data

All manuscripts must include a data availability statement. This statement should provide the following information, where applicable:
- Accession codes, unique identifiers, or web links for publicly available datasets
- A list of figures that have associated raw data
- A description of any restrictions on data availability

The summary results generated during this study are available at the AMP-T2D Portal, http://t2d.hugeamp.org/.

Field-specific reporting

Please select the one below that is the best fit for your research. If you are not sure, read the appropriate sections before making your selection.

- [x] Life sciences
- [ ] Behavioural & social sciences
- [ ] Ecological, evolutionary & environmental sciences

For a reference copy of the document with all sections, see nature.com/documents/nr-reporting-summary-flat.pdf

Life sciences study design

All studies must disclose on these points even when the disclosure is negative.

Sample size	N=26,807 for fasting glucose and N=23,211 for fasting insulin was used based on availability of data.
Data exclusions	Pre-established exclusion criteria included: individuals with diabetes; in order to understand the genetic basis of glycemia.
Replication	Replication of novel variants in additional populations were undertaken and results are reported in the manuscript. For variants where we did not find statistically significance this could be due to insufficient power or a type 1 error.
Randomization	Participants were not randomized. Genetic association analyses were adjusted for standard covariates including genetic relatedness, principal components, age, gender and body mass index.
Blinding	Blinding did not occur since this was not an intervention trial.

Reporting for specific materials, systems and methods

We require information from authors about some types of materials, experimental systems and methods used in many studies. Here, indicate whether each material, system or method listed is relevant to your study. If you are not sure if a list item applies to your research, read the appropriate section before selecting a response.

Materials & experimental systems

n/a	Involved in the study	
☒	☐	Antibodies
☒	☐	Eukaryotic cell lines
☒	☐	Palaeontology and archaeology
☒	☐	Animals and other organisms
☐	☒	Human research participants
☒	☐	Clinical data
☒	☐	Dual use research of concern

Methods

n/a	Involved in the study	
☒	☐	ChIP-seq
☒	☐	Flow cytometry
☒	☐	MRI-based neuroimaging

Human research participants

Policy information about studies involving human research participants

Population characteristics	Participants in this study were individuals without diabetes, 65% or 50% female, average age of 54+/-14 years or 50+/-17 years for fasting glucose or fasting insulin, respectively. Participants represented five self-reported race/ethnicity groups; 55% European, 28% African, 7% Hispanic/Latinx, 6% Asian, and 4% Samoan.
Recruitment	Results reported in this manuscript involved participants from 15 unique studies each with their own criteria and recruitment strategies but in general had the goal of understanding cardiometabolic diseases.
Ethics oversight	Each study's institutional review boards provided ethical oversight; and each analyst who had access to phenotypic and genotypic data had ethical oversight and review from their institution's to conduct the analysis of data used in this manuscript.

Note that full information on the approval of the study protocol must also be provided in the manuscript.
